# Mechanism of impaired consciousness in absence seizures: a cross-sectional study

**DOI:** 10.1016/S1474-4422(16)30295-2

**Published:** 2016-12

**Authors:** Jennifer N. Guo, Robert Kim, Yu Chen, Michiro Negishi, Stephen Jhun, Sarah Weiss, Jun Hwan Ryu, Xiaoxiao Bai, Wendy Xiao, Erin Feeney, Jorge Rodriguez-Fernandez, Hetal Mistry, Vincenzo Crunelli, Michael J. Crowley, Linda C. Mayes, R. Todd Constable, Hal Blumenfeld

**Affiliations:** 1 Yale University School of Medicine, Department of Neurology, Museum Avenue, Cardiff CF10 3AX, UK; 2 Department of Diagnostic Radiology, Museum Avenue, Cardiff CF10 3AX, UK; 3 Department of Child Study Center, Museum Avenue, Cardiff CF10 3AX, UK; 4 Department of Neuroscience, and, Museum Avenue, Cardiff CF10 3AX, UK; 5 Department of Neurosurgery, New Haven, CT, Museum Avenue, Cardiff CF10 3AX, UK; 6 Department of School of Biosciences, Cardiff University, Museum Avenue, Cardiff CF10 3AX, UK

## Abstract

**Background:**

Absence seizures are brief episodes of impaired consciousness characterized by staring and behavioral arrest. The neural underpinnings of impaired consciousness and of the variable severity of behavioral impairment observed from one absence seizure to the next are not well understood. We therefore compared fMRI and EEG changes in absence seizures with impaired task performance to seizures in which performance was spared.

**Methods:**

Patients were recruited from 59 pediatric neurology practices including hospitals and neurology outpatient offices throughout the United States. We performed simultaneous electroencephalography (EEG), fMRI, and behavioral testing in children and adolescents aged 6 to 19 years with typical absence epilepsy. fMRI and EEG were analyzed using data-driven approaches without prior assumptions about signal time courses or spatial distributions. The main outcomes were fMRI and EEG amplitudes in seizures with impaired versus spared behavioral responses analysed by t-test. We also examined the timing of fMRI and EEG changes in seizures with impaired behavioral responses compared to seizures with spared responses.

**Findings:**

93 patients were enrolled between September 1, 2005 and January 1, 2013, and we captured a total of 1032 seizures in 39 patients. fMRI changes during seizures occurred sequentially in three functional brain networks previously well-validated in studies of normal subjects. Seizures associated with more impaired behavior showed higher fMRI amplitude in all three networks compared to seizures with spared performance. In the default-mode network fMRI, amplitude was 0·57 ± 0·26% for seizures with impaired and 0·40 ± 0·16% for seizures with spared behavioral responses (mean difference 017%; 95% CI: 0·11 to 0·23%; p < 0.0001). In the task-positive network, fMRI amplitude was 0·53 ± 0·29% for impaired and 0·39 ± 0·15% for spared seizures (mean difference 0·14%; 95% CI: 008 to 0·21%; p < 0.0001). In the sensorimotor-thalamic network, fMRI amplitude was 0·41 ± 0·25% for impaired and 0·34 ± 014% for spared seizures (mean difference 0 07%; 95% CI: 001 to 0·13%; p = 0.02). Seizures with impaired behavior also showed greater EEG power in widespread brain regions compared to seizures with spared behavior. Mean fractional EEG power in the frontal leads was 50·4 ± 15·2 for seizures with impaired and 24·8 ± 6·5 for seizures with spared behavior (mean difference 25·6; 95% CI: 210 to 30·3); middle leads 35·4 ± 6·5 for impaired, 13 3 ± 34 for spared seizures (mean difference 22·1; 95% CI: 20.0 to 24·1); posterior leads 41·6 ± 5·3 for impaired, 24·6 ± 86 for spared seizures (mean difference 170; 95% CI: 14·4 to 19·7); p < 00001 for all comparisons. Average seizure duration was longer for seizures with impaired behavior at 79 ± 66 s, compared to 3·8 ± 3.0 s for seizures with spared behavior (mean difference 4.1 s; 95% CI 3.0 to 5.3 s, p < 00001). However, larger amplitude fMRI and EEG signals occurred at the outset or even preceding seizures with impairment.

**Interpretation:**

Impaired consciousness in absence seizures is related to the intensity of physiological changes in established networks affecting widespread regions of the brain. Increased EEG and fMRI amplitude occurs at the onset of seizures associated with behavioral impairment. These findings suggest that a vulnerable state may exist at the initiation of some seizures leading to greater physiological changes and altered consciousness.

## Introduction

Consciousness depends on normal large-scale network function in the brain.^1-3^ Impairment of large-scale brain networks leads to disorders of consciousness such as coma, vegetative or minimally conscious states with broad deficits in behavioral responsiveness and higher cognitive performance.^4^ In neurological disorders, impairment of consciousness is defined on the basis of altered responses in many different tests together leading to overall dysfunction in alertness, attention, or awareness.^4-6^ Absence epilepsy is a relatively common disorder that affects 10 to 17% of children and adolescents with epilepsy^7^ presenting with brief episodes of impaired consciousness and generalized 3-4 Hz spike-wave discharge on electroencephalography (EEG). Absence seizures meet the definition of a disorder of consciousness by causing transient deficits on a broad range of tests including verbal, visuo-motor, auditory-motor, memory and attentional vigilance testing.^8^ Absence epilepsy is not benign and causes significant psychosocial disability;^7,9,10^ however, the mechanism for impaired consciousness in absence seizures is not known. Importantly, although all absence seizures involve similar-appearing spike-wave discharges on EEG, some impair consciousness whereas others spare consciousness even in the same patient.^11-12^ There are several possible explanations for why some absence seizures impair and others spare consciousness. One possibility is that both seizure types involve generalized brain networks, but that seizures that impair consciousness affect widespread networks more intensely than seizures which spare consciousness. A second possibility is that seizures that impair or spare consciousness may involve different focal regions of the brain.^13-16^ Prior work also suggests that longer absence seizures tend to cause more severe behavioral impairment than shorter seizures. Therefore, another question is whether longer duration causes worse impairment simply because there is more time for brain physiology to be altered. Alternatively, seizures with impaired behavior may be physiologically more severe from the outset.

To examine these questions, we tested behavioral responsiveness as an indicator of consciousness in a cohort of patients with typical childhood or juvenile absence epilepsy during EEG and functional magnetic resonance imaging (fMRI) of the brain. We then compared brain network involvement during seizures that impaired versus those that spared responsiveness.

## Methods

For additional details see Supplementary Methods (Figures S1-S7) in the Appendix.

### Study design and participants

All study procedures were done at Yale School of Medicine. Patients were recruited from 59 pediatric neurology practices throughout the United States or through Internet advertisements. Recruitment and data collection took place from January 2005 to September 2013 with no follow-up planned in the study design. Patients were selected by telephone screening and enrolled with the following inclusion criteria: age 6–19 years, diagnosis of childhood or juvenile absence epilepsy based on International League Against Epilepsy classification,^17^ and EEG with typical 3–4 Hz bilateral spike-wave discharges and normal background; as well as the following exclusion criteria: additional seizure types including myoclonic, tonic-clonic, or partial seizures, structural brain abnormalities, or other neurological disorders.

Yale University's Institutional Review Board provided ethics approval for all procedures, and all participants and families provided written informed assent and consent.

The outcomes in this study were behavioural responsiveness and amplitude of fMRI and EEG signals during absence seizures. We aimed to assess the relation between behavioural responsiveness during absence seizures and amplitudes of fMRI and EEG signals. Anti-epileptic medications were withheld for up to 48 hours prior to recordings as described previously^10,12,18,19^ to improve the likelihood of capturing ictal data and to mitigate medication effects. Procedures including hyperventilation, photostimulation, and sleep deprivation were not used due their potential effects on fMRI signal or subjects' level of attention.

Participants underwent two behavioural measures of attention during seizures, the continuous performance task (CPT) and repetitive tapping task (RTT).^8,11,12,19^ Letters were presented at one hertz on a screen using E-Prime 1· 1 (Psychology Software Tools, Pittsburgh, PA). For the CPT, subjects pressed a button each time they saw a target letter “X” out of a random letter sequence. In the similar but easier RTT, subjects pressed a button each time they saw any letter on the screen. For both CPT and RTT, 32- or 96-seconds of task were alternated with 32-seconds of fixation for a total run duration of 640 seconds. Patients performed 3-6 runs of task per session as tolerated. Some patients also underwent runs of fixation only without task during fMRI to help define the general timecourse of fMRI changes during absence seizures even without task.

fMRI was acquired on a 3-T scanner (Siemens Medical Systems, Germany) using parameters as previously reported and initial processing steps to standardize images to the same three-dimensional space.^10,19^ Quality of fMRI data was maintained by removal of runs or frames meeting criteria for signal-to-noise ratio and motion. In-scanner 32-lead EEG was acquired with carbon-wire electrodes and processed to remove scanner artifact.^12,19,20^ For out-of-scanner high-density EEG, we used a 256-lead cap with Netstation v4·2 software (Electrical Geodesics Incorporated, Inc.).

### fMRI analysis

Absence seizures show evolving fMRI signal changes before and after electrical activity on EEG.^19,21,22^ Therefore, our fMRI analyses used a data-driven approach and included a peri-ictal period from -30 to +58 seconds relative to seizure onset (defined as time 0). We first performed k-means clustering on the fMRI signal over time from a large number of seizures to obtain generalizable network regions involved in absence seizures. For each network, fMRI signal changes were plotted over time to obtain their characteristic blood oxygenation hemodynamic response function (HRF) to seizure activity. Statistical parametric mapping (SPM, http://www.fil.ion.ucl.ac.uk/spm) was then performed using HRFs specific for each network to investigate fMRI differences between the subset of seizures with impaired or spared behavioral performance.

### EEG analysis

EEGs were read by two reviewers and confirmed by an experienced epileptologist for the presence and timing of seizures. Seizure periods from high-density EEG recordings were transformed into the frequency domain using a Fourier transform. Because absence seizures are composed of low-frequency 3-4 Hz waves and high-frequency (> 10 Hz) spikes, analysis focused on the corresponding 2 5-4 Hz and 10-125 Hz ranges. EEG power during seizures divided by baseline (fractional power) for each lead was plotted on a head map and quantified in anterior, middle, and posterior regions for seizures with spared versus impaired performance. Timecourses of fractional EEG amplitude over each region were also plotted for spared versus impaired seizures.

### Statistical analysis

Statistical analysis was performed using MATLAB and SPM with the main outcome variables of correct response rate on behavioral testing, signal amplitude on fMRI, and signal amplitude on EEG during seizures. Because subjects could have seizures with impaired performance, spared performance, or both, we used seizures rather than subjects as the most logical unit of analysis. We also performed more stringent subject-based analyses for the smaller subset of patients who had seizures with both paired and spared performance. The main analyses combined results across the CPT and RTT behavioral tasks, but separate subgroup analyses of each task were also performed. The size of our study sample was determined based on previous similar studies in this population that reached statistical significance.^16,19,21,23^ Analyses were performed by two-tailed t-test with significance threshold p<0 05. For fMRI analyses, significance threshold was p < 005 with family-wise error correction for multiple comparisons. Spearman's correlation coefficient r was also calculated where indicated. Additional details can be found in the Supplementary Methods p. 3.

### Role of the funding source

The funders of the study had no role in study design, data collection, data analysis, data interpretation, or writing of the report. The corresponding author had full access to all the data in the study and had final responsibility for the decision to submit for publication.

## Results

**Figure 1:**
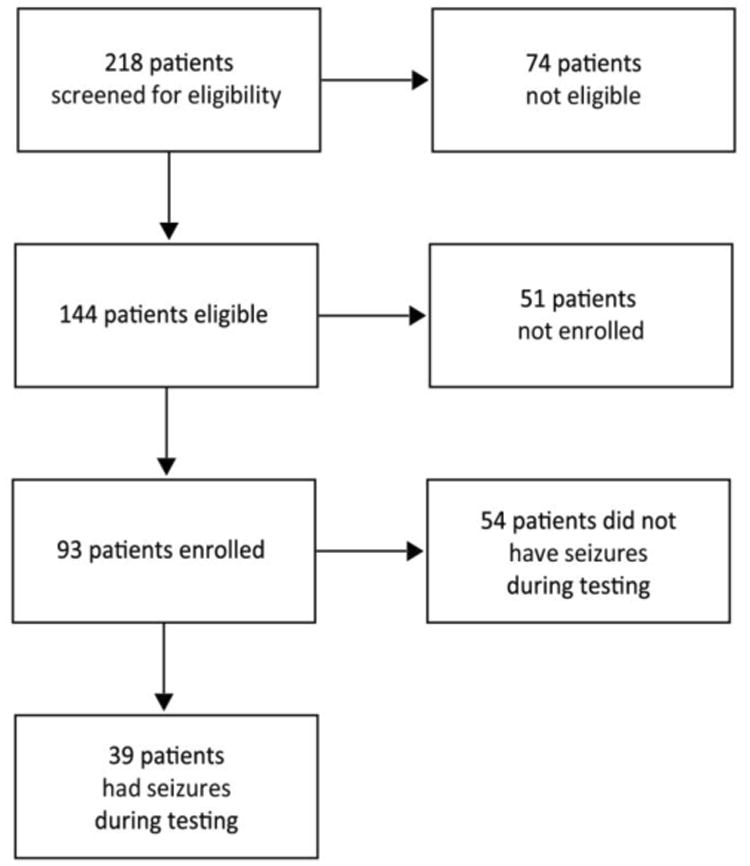
Study profile Patients were screened for eligibility by telephone (see Methods). Eligible patients underwent EEG-fMRI or high-density EEG without fMRI to obtain data during seizures.

We evaluated 218 patients for eligibility between January 1, 2005 and September 1, 2013. 93 patients were enrolled, of which 39 had absence seizures during testing (Figure 1, Table 1; 1032 seizures total). 23 of 39 (59%) subjects were female; mean age (± SD) was 9·9 ± 3·2 years and duration of epilepsy was 3·0 ± 2·3 years, not significantly different from patients without seizures during testing. However, patients with seizures were more commonly off medications prior to the study (Table 1) and had longer total testing time per subject (mean ± SD, 2·3 ± 1·8 versus 0·9 ± 0·7 hours; mean difference 1·4 hours, 95% CI 0·9 to 2·0; P < 0·0001. Of the 1032 seizures, 810 from 34 patients were used for general fMRI timecourse analysis (Figure 3) and fewer met strict exclusion criteria for analysis of behavior in relation to fMRI or EEG (205 seizures in 27 patients for fMRI in Figure 4; 56 seizures in 10 patients for EEG in Figure 5) (see Supplementary Methods p. 5 and 7 for details). No subjects experienced generalized tonic-clonic seizures or other adverse events during the period of time that medications were withheld for the study (see also Supplementary Methods p. 3).

**Figure 2:**
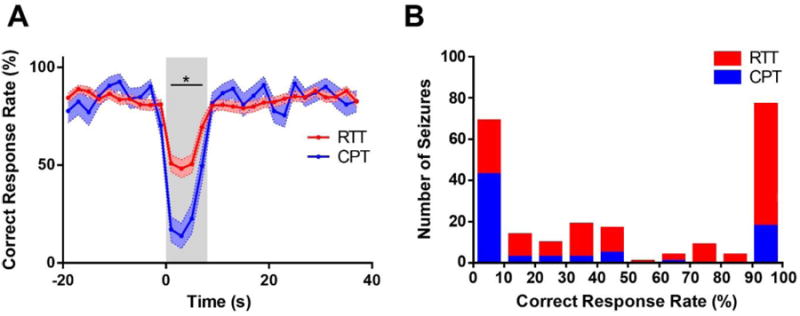
Behavioral performance on two tasks during seizures. (A) Timecourse of behavioural performance on the repetitive tapping task (RTT) and continuous performance task (CPT). Gray indicates seizure periods, normalized to mean seizure duration (8 seconds). Data are plotted in two-second time bins, and dotted lines indicate standard error of the mean (SEM) for each bin. (B) Bimodal distribution of task performance (% targets with correct response) for individual seizures with the two tasks.

Mean performance on both the CPT and RTT tasks was rapidly impaired at seizure onset (Figure 2A, time = 0; n = 74 seizures in 24 patients for CPT; n = 148 seizures in 32 patients for RTT), reached a trough, and then quickly returned to baseline at seizure end. Mean (± SD) correct response rate dropped from 82·9 ± 17·0% at baseline (interictal) to 58·3 ± 39·7% during seizures for RTT (mean percent difference 24.6, 95% CI 187 to 305, p <00001, calculated across seizures). Mean correct response rate for CPT dropped from 83·7 ± 23·8% interictally to 35·5 ± 44·1% during seizures (mean percent difference 48·2, 95% CI 400 to 56·4, p <00001, calculated across seizures). Subjects showed greater mean impairment during the more attentionally demanding CPT compared to RTT (Figure 2A). Mean ictal correct response rate for CPT was 35·5 ± 44.0%, compared to RTT at 58·3 ± 40.0% (mean percent difference 22·8, 95% CI 11·2 to 34·4, p = 00001, again calculated across seizures). Behavioral timecourses in Figure 2A include baseline data surrounding additional seizures (136 seizures in 28 patients for CPT, 75 seizures in 26 patients for RTT) where task targets occurred immediately before or after but not during seizures.

Importantly, responses were spared during some of the seizures for both tasks (Figure 2B). Ictal performance showed a bimodal distribution for both tasks, with the majority of seizures having either low or high correct response rates. This provided a natural division in the data allowing us to define “impaired” seizures as those with < 25% and “spared” seizures as those with > 75% response rates to behavioral targets. No significant demographic differences were observed between patients with spared or impaired seizures (Table 1); seizures associated with both spared and impaired behavior could occur within the same individual (Table S1). Age was not significantly correlated with ictal performance for either task (CPT r = 0.19, RTT r = -0.34, p > 0.05 for both correlations). To ensure the bimodal distribution in Figure 2B was not entirely due to seizures in which only one target occurred (therefore with 0% or 100% as only possible results), we reanalyzed the data excluding these seizures (see Figure S8). Because the overall timecourse and distribution of impairment were similar on the two tasks (aside from worse average performance during seizures on CPT (see Figure 2A), data from the two tasks were combined for subsequent analyses by sorting seizures into those showing impaired or spared performance regardless of task. Separate results by task are in the Supplementary Appendix p. 6 and 10.

**Figure 3:**
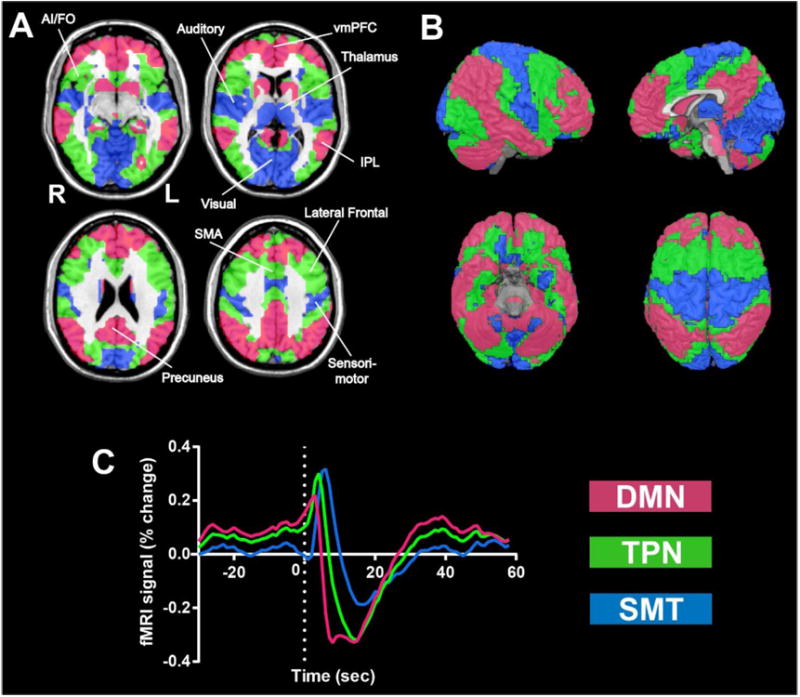
Sequential involvement of known large-scale networks during seizures. (A) Axial brain slices with parcellation of functional MRI activity into three distinct networks during seizures using k-means clustering. These networks encompass the default-mode network (DMN), task-positive network (TPN), and primary sensorimotor-thalamic network (SMT). (B) The three networks on whole brain surface renderings. (C) Distinct timecourses of fMRI signals for each of the three networks as a percent change of total signal (0 s = seizure onset). n = 810 seizures in 34 patients. Anterior insula/frontal operculum (AI/FO); Ventral medial prefrontal cortex (vmPFC); Inferior parietal lobule (IPL); Supplementary motor area (SMA).

fMRI timecourse analysis of seizures showed a widespread, evolving pattern of activations and deactivations that persisted long after electrical activity stopped on EEG as in prior work (Figure 3C).^19,21,22^ K-means clustering was performed on the mean fMRI timecourse across all 810 seizures to determine regions showing similar timecourses based on correlation between voxels over time (See Supplementary Methods, p. 8). This approach provided regions generalizable across seizures and revealed sequential involvement of three well-known networks^24^: the default-mode network, task-positive network, and primary sensorimotor cortices and thalamus (Figure 3A and 3B). Because the clustering included the entire brain, each network extended somewhat beyond its traditional boundaries. Thus, the default-mode network, in addition to the ventral medial frontal, posterior cingulate/precuneus, lateral parietal, lateral and medial temporal cortex, also extended to the basal ganglia and other cortical/subcortical regions. The task-positive network included top-down attention areas such as the lateral frontal and parietal cortex, as well as salience regions such as the anterior insula/frontal operculum, dorsal medial frontal cortex and supplementary motor area. Finally, the primary sensorimotor network included occipital (visual), dorsolateral temporal (auditory), Rolandic (somatosensory/motor) cortex, and thalamus. These networks showed distinct, sequential fMRI responses during seizures (Figure 3C). The default-mode network showed small early activations followed by large deactivations. Next, the task-positive network showed a more biphasic response, with early activations roughly matched in amplitude by later deactivations. Finally, the primary sensorimotor areas and thalamus showed activations followed by relatively smaller late deactivations (Figure 3C). Clustering the data into more than three networks was possible but yielded less cohesive clusters (Figures S3, S4) so we focused on these three networks for the remaining analyses.

**Figure 4:**
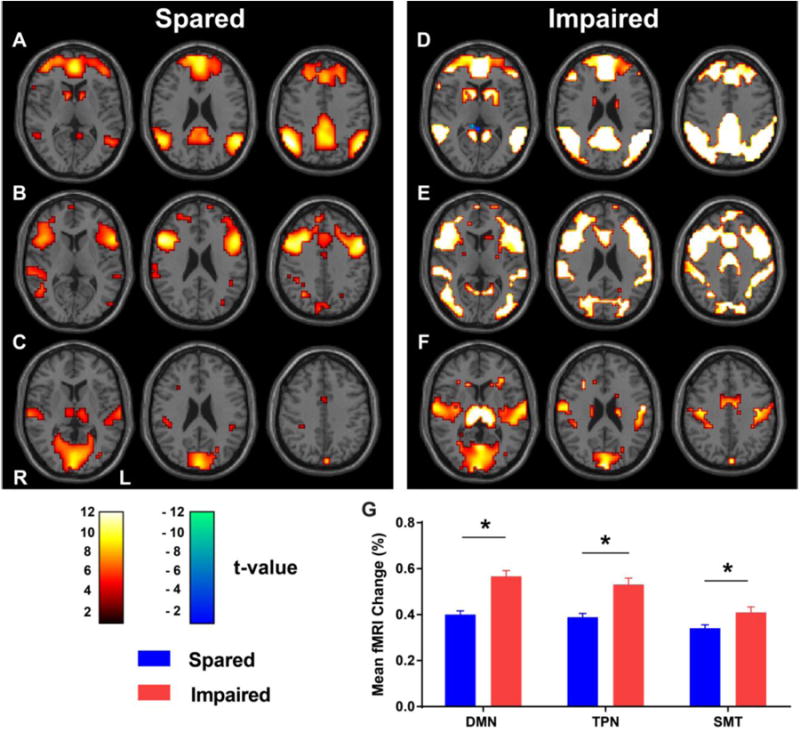
Larger fMRI signals in all three networks during seizures with impaired task performance. (A-C) Axial brain t-maps with fMRI signals for seizures with spared performance in the default-mode network (A, DMN), task-positive network (B, TPN), and primary sensorimotor-thalamic network (C, SMT). (D-F) Corresponding t-maps for seizures with impaired performance. Hot colors indicate brain regions with significant fMRI changes in the same direction as the network-specific HRFs. Cool colors indicate changes in the opposite direction. (G) Mean fMRI percent change across seizures in each network. n = 93 spared seizures in 17 patients, and 112 impaired seizures in 22 patients.

Seizures with impaired or spared behavior showed similar widespread involvement of all three networks on fMRI (Figure 4). However, the intensity of fMRI signals in all three networks was greater with “impaired” seizures compared to “spared” seizures. Mean percent differences were as follows: default-mode network 017% (impaired: 0·57 ± 0·26%; spared: 0·40 ± 0·16%; 95% CI: 0·11 to 0·23%, p < 00001); task-positive network 0·14% (impaired: 053 ± 029%; spared: 039 ± 015%; 95% CI: 008 to 021%, p < 00001); sensorimotor-thalamic network 007% (impaired: 0·41 ± 0·25%; spared: 0·34 ± 0·14%; 95% CI: 001 to 0·13%, p = 0.02)). See also Figure S9 for t-maps directly contrasting seizures with impaired versus spared performance. These results were confirmed in separate subgroup analyses for the two behavioral tasks CPT and RTT (Figures S10, S11) and were robust to different choices for behavioral cutoffs other than <25% and >75% (Figures S12, S13). A more stringent analysis on the smaller sample of patients (n = 11) who had both seizures with impaired and spared performance showed larger amplitude in seizures with behavioral impairment although this did not reach statistical significance (Figure S14).

**Figure 5:**
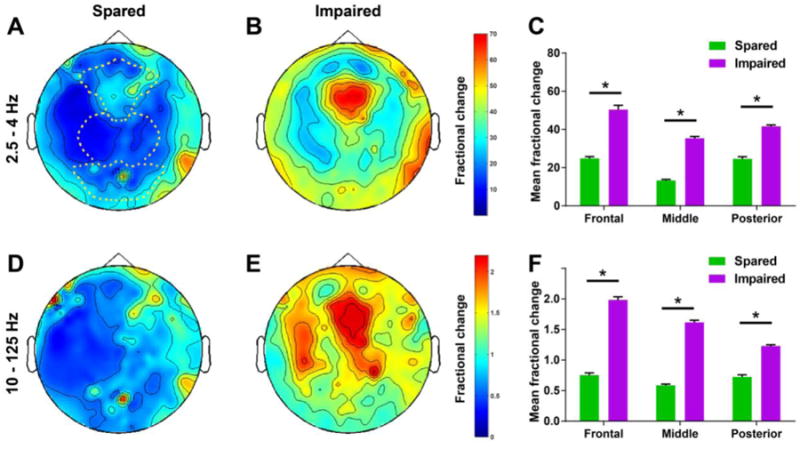
Greater EEG amplitude in widespread brain regions during seizures with impaired task performance. (A-B) Head maps of 256 channel high-density EEG power in the 2·5-4 Hz frequency range, representing the wave components of spike-wave discharges for seizures with spared (A) or impaired (B) task performance. (C) Mean fractional EEG power in the 2·5-4 Hz frequency range for seizures with spared versus impaired performance. (D-E) Maps of EEG power in the 10-125 Hz frequency range (spike components of spike-wave discharges) for spared (D) and impaired (E) seizures. (F) Mean fractional EEG power in the 10-125 Hz frequency range for seizures with spared versus impaired performance. Color scale bars are EEG power during seizures divided by baseline power prior to seizures (fractional power). The top color bar is for panels (A) and (B), and bottom bar is for (D) and (E). Regions used for analysis in (C) and (F) (Frontal, Middle, Posterior) are shown by yellow dashed lines in (A). * p < 0·0001. Error bars are SEM. n = 30 spared seizures in 5 patients, and 26 impaired seizures in 8 patients.

EEG showed broadly distributed 3-4 Hz spike-wave discharges with a frontal amplitude predominance (see Figures S1, S2) as in previous studies^15^ and similar overall distribution pattern for seizures with spared or impaired performance (Figure 5 A, B, D, E). However, seizures with impaired task performance demonstrated greater EEG power in widespread regions compared to seizures with spared task performance (Figure 5C, F). Impaired seizures showed greater amplitude in the frequency range of both waves (2·5-4 Hz and spikes (10-125 Hz) in frontal to occipital regions. Mean differences in fractional EEG power were as follows for waves: Frontal leads 25·6 (impaired: 50·4 ± 15·2; spared: 24·8 ± 6·5; 95% CI: 210 to 30·3); Middle leads 22·1 (impaired: 35·4 ± 6·5; spared: 13 3 ± 3·4; 95% CI: 20·0 to 24·1); Posterior leads 170 (impaired: 41·6 ± 5·3; spared: 24·6 ± 8·6; 95% CI: 14·4 to 19·7); and as follows for spikes: Frontal leads 123 (impaired: 198 ± 0·36; spared: 0·75 ± 0·26; 95% CI: 111 to 136); Middle leads 103 (impaired: 1·62 ± 0·24; spared: 0·59 ± 0·14; 95% CI: 0·95 to 111); Posterior leads 0·51 (impaired: 123 ± 015; spared: 0·72 ± 0·27; 95% CI: 0·43 to 0·59), with p < 00001 for all comparisons. See Figure S15 for individual seizure examples. Results were confirmed in separate subgroup analyses for the two behavioral tasks (Supplementary Figures S16, S17), using a different behavioral cutoff (Figure S18), and with the small subset of three patients who had both seizures with impaired and spared performance (Figure S19).

**Figure 6:**
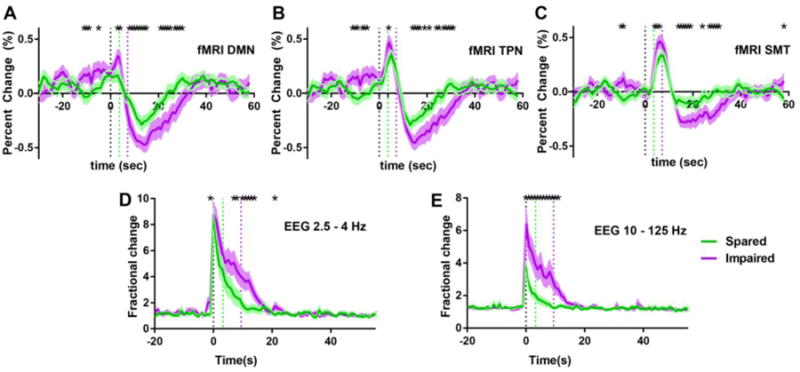
Greater fMRI and EEG amplitude from the outset or preceding seizures with impaired behavior. (A-C) Timecourse of percent fMRI signal changes in the default mode (A), task positive (B), and sensorimotor-thalamic (C) networks for seizures with spared (green) and impaired (purple) task performance. (D-E) Timecourse of EEG amplitude fractional change (seizure divided by baseline) over time for low (2·5-4 Hz, D) and high (10-125 Hz, E) frequency ranges representing waves and spikes of seizures. (* p < 0 05). Timecourses are aligned to seizure onset (time = 0s) showing mean and SEM of signals. Data for (A-C) are from the same patients and seizures as Figure 3, and data for (D-E) are from the same patients and seizures as Figure 4. Dotted vertical lines indicate seizure onset (black) or offset (green and purple for seizures associated with spared or impaired performance, respectively).

Seizure duration was longer in the behaviorally impaired seizures (79 ± 66 s; mean ± SD) compared to the behaviorally spared seizures (3·8 ± 3·0 s) (p < 0·0001; mean difference 4.1 s; 95% CI 3.0 to 5.3 s). To determine the timing of physiological differences between seizures with impaired versus spared task performance we examined the timecourses of fMRI and EEG changes (Figure 6). fMRI showed larger amplitude from the outset in impaired seizures in all three identified networks (Figure 6 A-C). Interestingly, the larger fMRI amplitude in impaired seizures actually preceded seizure onset by about ten seconds. Later fMRI increases as well as decreases were also larger in seizures with impaired performance. Similarly, EEG measurements showed greater amplitude from the outset of seizures with impaired performance (Figure 6 D-E). EEG amplitude for both waves (2·5-4 Hz) and spikes (10-125 Hz) diverged at or just prior to the time of seizure onset. Thus seizures with impaired performance showed more severe physiological changes on both fMRI and EEG at or even before seizure onset.

## Discussion

Using 1032 seizures captured from 39 patients with typical childhood or juvenile absence epilepsy, we found that the overall intensity of seizures correlates with behavioral impairment. This relationship held for seizure amplitudes on both fMRI and EEG in widespread regions of the brain, was present from the outset of seizures, and extended to tasks of varying difficulty. These results represent a fundamental shift from prevailing views of impaired consciousness in absence seizures, which hold that focal brain involvement^8,13,15^ or prolonged seizure duration^8^ determine the severity of impaired consciousness. Our data present a very different mechanism for impaired consciousness, demonstrating instead that: seizures with impairment have greater physiological intensity in widespread networks including most of the brain, not only in focal areas; and seizures with impairment are more physiologically intense at seizure initiation, not just at later times during seizures.

Recent theories of normal and disordered consciousness have recognized the importance of large-scale information integration or of a global neuronal workspace necessary for the emergence of consciousness.^1,3^ Neuroenergetic considerations also emphasize the overall function of widespread brain networks. However, it has long been recognized that absence seizures do not affect the brain homogenously and that some regions, particularly the midline frontal cortex, are more intensely involved than others on EEG.^15^ fMRI studies have also shown regional heterogeneity that varies from one patient to the next.^16,22^ Animal models suggest that spike-wave discharges arise from focal regions of enhanced cortical excitability.^14,25^ Our present findings demonstrate that although seizures may be more intense in some regions than others, the difference between seizures that do or do not impair consciousness is not confined to local regions, but rather affects virtually the entire brain. This supports and extends previous work which proposed that impaired consciousness in absence seizures is caused by default mode suspension together with reduced perception through sensory inputs, as well as impaired function of the thalamus, frontal regions and the insulae^23^. Thus, although seizures may have regional heterogeneity, our findings support a model in which global rather than local differences between seizures are related to impaired consciousness.

Longer duration of spike-wave discharges on EEG has been associated with more severe behavioral impairment^8^ although exceptions clearly exist.^8,11^ In agreement with a recent study^26^, fMRI amplitude was linearly related to spike-wave duration (Figure S7). An important question is whether longer seizures cause more severe effects simply because there is more time for longer seizures to induce physiological changes in the brain. However, because we incorporated this relation into our model, differences in fMRI signals between seizures with spared versus impaired task performance (Figure 4) were not simply due to spike-wave duration. Alternatively, longer and more behaviourally impaired seizures might be more physiologically intense from the outset. Interestingly, some previous studies have shown that fMRI changes can precede seizures by several seconds,^19,21,22^ and our results identify early fMRI changes preceding seizures with behavioural impairment, but not when behaviour is spared. Additionally, we noted early differences in EEG signals between seizures with impaired versus spared task performance. These results suggest that greater behavioural impairment from longer seizures is not simply due to gradual worsening of abnormal physiology during the course of the seizure, but rather that some seizures have more severe physiological abnormalities at or before seizure onset. This raises the intriguing possibility that a vulnerable state occurs at the initiation of some seizures in which an unknown combination of intrinsic brain fluctuations and seizure initiation mechanisms interact to generate more physiologically and behaviorally severe seizures.

Whole-brain parcellation of the fMRI timecourse in absence seizures revealed three well-described networks, each with a unique hemodynamic response. These include sequential involvement of the default-mode, task-positive, and primary sensorimotor-thalamic networks. Therefore even pathological states such as absence seizures may acquiesce to established network boundaries. This result complements evidence from normal subjects whereby established cortical networks persist across states from rest to task processing,^24^ and in normal loss of consciousness in transitioning from wakefulness to sleep.^27^ Successful task performance requires coordination of multiple cognitive processes. Primary visual and motor cortices are involved in sensory input and motor output, while attention initiation and maintenance involves a wider collection of areas including dorsal and ventral fronto-parietal circuits and the default-mode network. However, while temporally covarying activity across regions is thought to reflect processing of mutual information under normal task conditions, the hypersynchronous state of the brain in these same networks during absence seizures may paradoxically impair normal functional connections and behavior.

To investigate the full spatio-temporal profile of fMRI changes during seizures, we used a data-driven approach without prior assumptions about the signals. To analyze EEG signals, we likewise avoided models or assumptions and simply examined amplitude mapped over the recording surface. The strong convergence between fMRI and electrophysiological results in our data allays possible concerns that in some circumstances fMRI may not accurately reflect underlying neuronal activity.^25^ Notably, both fMRI and EEG demonstrated early and widespread increased amplitude in seizures with behavioral impairment. One additional potential factor to consider is the effect of breath-holding during seizures on fMRI amplitude^28^, which could be addressed by measuring breathing during fMRI in future studies.

An important remaining question is why fMRI and EEG signal increases and decreases are larger in some seizures, namely those with more severe behavioral impairment. The amplitude of fMRI signals under normal conditions^29,30^ and during spike-wave seizures^14,25^ is related to the number of neurons involved in a given volume of brain and the intensity of their electrical activity. EEG amplitude is also related to the intensity and the synchrony of neuronal electrical activity. We postulate that seizures with more severe behavioral impairment engage a larger number and/or more intense activity in neurons in the seconds preceding obvious EEG onset, which may then cause larger and longer-lasting fMRI and EEG changes throughout the brain during and following seizures. Our study is unable to demonstrate whether particular areas of the networks rather than entire networks cause impairment in consciousness, and the possibility remains that some specific region or regions are more important than others. However, we show widespread brain involvement in absence seizures as well as correlation of behavioral impairments with disruptions in each network. Our cross-sectional study using noninvasive measurements on human subjects is intrinsically limited in testing direct causal relationships between brain states and behavior. The fundamental mechanisms determining seizure severity will require further investigation, perhaps best performed in experimental models where direct neuronal recordings can be obtained.

Absence seizures are not benign, and cause substantial behavioral deficits in many children living with this disorder.^8,9^ Our observation that larger amplitude fMRI and EEG changes occur in widespread networks early during initiation of more severe seizures may have broader implications for other seizure types and for brain-state transitions in general. These findings suggest that future work should investigate the physiology of these early, widespread changes in brain networks. Crucially, understanding the mechanisms regulating absence and other seizure severity may ultimately provide new opportunities to intervene therapeutically and protect patients from deficits associated with seizures.

## Supplementary Material

supplement

## Figures and Tables

**Table 1:** Clinical and demographic characteristics

Group	Number of seizures	Number of patients	Number of females (%)	Age (years, mean ± SD)	Duration of epilepsy (years, mean ± SD)	Number of patients off medication pre-study (%)
Patients without seizures	0	54	27 (50%)	10·6 ± 3·7	4·0 ± 3·7	6 (11%)
Patients with seizures	1032	39	23 (59%)	9·9 ± 3·1	3·0 ± 2·5	14 (36%)
Comparison*			P= 1·000	P=0·329	P= 0·128	P=0 005
Spared seizures total	139	21	11 (52%)	10·6 ± 3·2	2·9 ± 0·9	6 (29%)
Impaired seizures total	160	27	17 (63%)	100 ± 31	3·2 ± 2·6	11 (41%)
Comparison*			P=0·560	P=0·541	P=0·691	P=0·544
Spared seizures fMRI	93	17	8 (47%)	10·7 ± 3·3	3·3 ± 21	5 (29%)
Impaired seizures fMRI	112	22	16 (73%)	10·2 ± 3·3	3·8 ± 2·3	8 (36%)
Comparison*			P=0 184	P=0·631	P=0·554	P=0·740
Spared seizures HD-EEG	30	5	2 (40%)	13·5 ± 3·6	3·3 ± 2·9	4 (80%)
Impaired seizures HD-EEG	26	8	5 (63%)	110 ± 4·5	2·8 ± 2·8	5 (63%)
Comparison*			P=0·592	P=0·303	P=0·752	P= 1·000

Spared and impaired seizures total groups include all seizures used for analyses in Figures 2, 4, 5 and 6. Spared and impaired seizures fMRI or HD-EEG groups include all seizures used for Figures 4, 5 and 6.

* Two-tailed t-test was used for age and duration of epilepsy; Fisher's exact test was used for % female and % off medication pre-study.

See also Table S1 for additional characteristics of individual patients with seizures.
