# Supplementary material for: Mechanism of impaired consciousness in absence seizures: a cross-sectional study
Source: Lancet Neurol. Author manuscript; Available in PMC 2017 Dec 1. (PMC5504428; doi:10.1016/S1474-4422(16)30295-2)
Supplement: supplement [file NIHMS871899-supplement.docx]

­

Supplementary Online Material for:

**Mechanism of impaired consciousness in**

**absence seizures: a cross-sectional study**

Jennifer N. Guo^1^, Robert Kim^1^, Yu Chen^1^, Michiro Negishi^2^, Stephen Jhun^1^, Sarah Weiss^1^, Jun Hwan Ryu^1^, Xiaoxiao Bai^1^, Wendy Xiao^1^, Erin Feeney^1^, Jorge Rodriguez-Fernandez^1^, Hetal Mistry^1^, Vincenzo Crunelli^6^, Michael J. Crowley^3^, Linda C. Mayes^3^, R. Todd Constable^2^ and Hal Blumenfeld^1,4,5^

Yale University School of Medicine, Departments of ^1^Neurology, ^2^Diagnostic Radiology, ^3^Child Study Center, ^4^Neuroscience, and ^5^Neurosurgery, New Haven, CT
^6^School of Biosciences, Cardiff University, Museum Avenue, Cardiff CF10 3AX, UK

Correspondence to: Hal Blumenfeld, MD, PhD

Yale Depts. Neurology, Neuroscience, Neurosurgery

333 Cedar Street, New Haven, CT 06520-8018

Tel: 203 785-3865 Fax: 203 737-2538

Email: [hal.blumenfeld@yale.edu](mailto:hal.blumenfeld@yale.edu)

**Table of Contents**

Supplementary Methods ………………………………… 3

Participants ………………………………… 3

Study procedures ………………………………… 3

Behavioral tasks and analysis ………………………………… 4

fMRI and EEG data acquisition ………………………………… 5

EEG analysis ………………………………… 5

fMRI analysis ………………………………… 6

Supplementary Figures S1-S19 ………………………………… 11

Supplementary Table S1 ………………………………… 32

References ………………………………… 34

## Supplementary Methods

**Participants**

All procedures were approved by the Yale University Institutional Review Board, and all participants and families provided written informed assent and consent. From January 2005 to September 2013, 93 subjects aged 6-19 years with typical childhood or juvenile absence epilepsy were enrolled. Participants were referred by their neurologists or responded to advertisements posted on the Internet. Inclusion criteria were as follows: 1. Clinical diagnosis of childhood or juvenile absence epilepsy based on International League Against Epilepsy classification(1); 2. EEG with typical 3–4 Hz bilateral spike-wave discharges and normal background activity as verified by clinical EEG report or EEG data; 3. age 6–19 years. Exclusion criteria were as follows: 1. Additional seizure types including myoclonic, tonic-clonic, or focal seizures; 2. structural brain abnormalities; and 3. other neurological disorders. Parents of all subjects underwent an intake history prior to participation to confirm eligibility including the following information: current patient age, age of onset, patient or parent description of episodes (duration, behaviors, experiences, auras, post-ictal changes), any convulsive or myoclonic seizures ever, seizure frequency at onset and currently, time of day most episodes occur, any known precipitants (photic stimulation, hyperventilation, sleep deprivation, stress, missed medications), current medications, when seizure medications were started and whether or not effective, previous medications tried, any other medications, and any other medical or neurological disorders. Past EEG records were obtained for all patients as a requirement for participation. When available, office records from referring pediatric neurologists were also obtained. All clinical information was then reviewed by an experienced epilepsy neurologist to confirm the diagnosis of childhood or juvenile absence epilepsy prior to participation. Of note subjects were not excluded based on parental report of early or late onset age if all other clinical features including EEG were typical of childhood or juvenile absence epilepsy.

Of the 93 patients tested, 39 had EEG-confirmed absence seizures during testing and were used for analysis. All analyses below apply to these 39 patients who had EEG-confirmed absence seizures during testing.

**Study Procedures**

Prior to study sessions, subjects underwent a practice session during a separate visit to become familiarized with the behavioral tasks and the MRI environment and sounds in a mock scanner. Feedback was given to subjects and parents to improve cooperation on the day of the scans and subjects were instructed to practice lying still in the days preceding the MRI scan, while parents rated their movement and provided feedback.

On subsequent study days, subjects underwent simultaneous EEG and fMRI recordings, and a subset also underwent a separate 256-lead high-density EEG outside of the scanner. Each study session day consisted of setup (approximately 40 minutes) followed by EEG-fMRI (approximately 40 minutes). In subjects who also performed high-density EEG additional setup (approximately 30 minutes) and testing (approximately 40 minutes) was performed. Number of testing session days per subject generally ranged from one to four, with an overall average of 1·5 ± 0·2 hours (mean ± SEM) of total testing time per subject. Subjects typically participated over the weekend on one or both weekend days in order to not miss school. Anti-epileptic medications were withheld up to 48 hours prior to the recordings as described previously (2-5) to improve the likelihood of capturing ictal data and to mitigate medication effects. This procedure was approved by our human subject Institutional Review Board as described in previous studies and has not caused any adverse effects (2-5). In particular, none of the study subjects had significant clinical worsening of their seizures or emergence of secondarily generalized tonic-clonic seizures or other new seizure types as a result of temporarily withholding their anti-epileptic medications for the study. Adverse effects were monitored on each day that subjects were off medications by phone calls from an epilepsy-trained neurologist to the families of participants with standard questions on the frequency, type of seizures and any other questions or concerns. The neurologist also remained available by cell phone to the families after participating in the study. Other procedures such as hyperventilation, photostimulation, and sleep deprivation were not used due their potential effects on the fMRI signal or subjects’ vigilance performance during behavioral tasks. Prior to the study, 28·2% of patients were not on medication, 41·0% were on ethosuximide, valproic acid, or lamotrigine, and the remainder were on a combination of medications or modified diet.

Because some patients had seizures during only the EEG-fMRI or high-density EEG sessions, or only at times when no behavioral task targets occurred, the number of subjects included in the analyses varied for each figure (see sample sizes in figure legends, Table 1 and Supplementary Table S1). We captured a total of 1032 seizures by either EEG-fMRI or high-density EEG. 810 of these seizures were seen on fMRI and were usable for general time course analysis (Figure 3). The rest were captured out of the scanner as part of the high-density EEG protocol. Of the 1032 seizures included in the study, 222 were captured while patients performed the tasks rather than during a period of fixation and also met strict exclusion criteria for movement or signal-to-noise and behavioral analysis time course exclusion criteria explained in more detail below (Figure 2A,B). 311 seizures were captured in the fMRI scanner with behavioral targets during seizure and of these 205 were classified as having either impaired or spared responses (Figure 4) and the remainder were included where intermediate behavioral performance was also considered (Figures S12, S13). 56 seizures (66 if including seizures with intermediate performance) were captured during high-density EEG with targets during seizure (Figures 5 and S18). Also as explained in the individual sections that follow, some seizures were excluded from EEG-MRI or high-density EEG analysis, for example, due to movement or other artifact but could still be included in behavioral analysis for Figure 2A,B. Conversely, some seizures were excluded from Figure 2A and 2B behavioral analysis due closely-spaced seizures with large behavioral fluctuations in overlapping baseline time windows but included in the fMRI model (where effects on the baseline signal were much smaller at <1%) in order to keep as much data as possible.

Behavioral tasks and analysis

Subjects performed two behavioral tasks as well as passive fixation during EEG and fMRI acquisitions. The behavioral tasks used have been well validated as measures of responsiveness during absence seizures in previous studies (5-8).Tasks were generated using E-Prime 1·1 (Psychology Software Tools, Inc. Pittsburgh, PA), and all visual stimuli were presented on a rear projection screen viewed by a mirror mounted on the head coil. For the continuous performance task (CPT), subjects were instructed to press a button each time they saw a target letter ‘X’ out of a random sequence of letters (A B C D E F H I L M N O T X Y Z) presented at one per second. The target letter ‘X’ was presented on average 25% of the time. In the similar but slightly easier repetitive tapping task (RTT), subjects were instructed to press a button for each letter presentation and no ‘X’ appeared in the sequence. Letters for both tasks were displayed for 250 ms at a rate of one per second. For both CPT and RTT, task blocks of either 32 s or 96 s duration were used, with 32 seconds of fixation in between blocks for a total behavioral run duration of 640 s. Tasks were alternated between runs and patients performed 3-6 runs of task per session as tolerated. Some patients also underwent identical runs of fixation only without task. Of the 39 subjects who had absence seizures during testing, 38 underwent CPT, 38 underwent RTT and 12 underwent fixation only without task. Of note, the number of seizures per unit time was not significantly different during task blocks vs fixation blocks with CPT (p=0·58) or with RTT (p=0·89).

Letter presentations and responses were recorded using the E-prime program; responses were also recorded on a separate EEG channel to verify synchronization between behavioral and EEG data acquisition. Analysis of behavioral data was performed using in-house code written on the MATLAB platform (MathWorks, Natick, MA). Omission rate on task (number of missed responses/number of targets) was calculated for each seizure and for the interictal periods of each run. Runs with an overall greater than 50% interictal omission error rate were omitted from behavioral analysis, as were sustained periods of no performance likely due to sleep or drowsiness. Correct responses for CPT were defined as occurring between 120-1000 ms after letter onset, and for RTT between 0-1000 ms after letter onset. Although occasionally patients had very delayed responses occurring >1000 ms after letter onset, these were rare events; therefore including them as correct responses did not significantly change the results (data not shown).

For group behavioral analysis (Figure 2A), seizure onset and end times were aligned, and the ictal periods were normalized to the average seizure duration as described previously(5). To accomplish this, ictal time course data for seizures of variable duration were linearly scaled to the average seizure duration of eight seconds. Temporal scaling was not applied to the pre-ictal or postictal periods, which were simply aligned to seizure onset and end times respectively. Ictal time course normalization was only performed for display of behavioral time courses in Figure 2A, but was not used for fMRI or EEG data. Previous work has shown that simple temporal scaling does not affect the major results when analyzing absence seizure group data time courses(5). Correct response rates were then calculated in two second time bins. Because of prior work showing seizure-related fMRI changes preceding and following seizures(5, 9, 10) we included data from up to 20 seconds before seizure onset and 30 seconds after seizure end in the behavioral time course analyses. Timepoints in the analysis window (-20 s from seizure onset, +30 s from seizure offset) that overlapped with the analysis window of a precedent or subsequent seizure were excluded from the analysis window for both seizures. We then compared overall performance in the ictal periods between the CPT and RTT tasks, as well as for both tasks compared to the interictal period using two-tailed t-tests.

fMRI and EEG data acquisition

fMRI data were acquired on a 3 Tesla Magnetom Trio scanner (Siemens Medical Systems, Germany) using a standard whole-head coil. Foam padding was used to secure EEG leads and reduce head motion. Anterior to posterior commissure (AC-PC) aligned, axial T1-weighted anatomical images were obtained (spin-echo sequence, repetition time = 300 ms, echo time = 2·47 ms, matrix size = 256 × 256, 25 slices, slice thickness 6 mm, field of view = 22 cm). Functional images where then acquired in the same image planes (echo-planar imaging sequence, repetition time = 1550 ms, echo time = 30 ms, flip angle = 80°, matrix size = 64 × 64). Each functional run consisted of 416 whole brain volumes acquired over 10 minutes and 44·8 seconds of scanning (4·8 s beyond the end of the behavioral run).

EEG data were acquired simultaneously with fMRI (EEG-fMRI) during all MRI scans. In addition, a subset of patients underwent separate out-of-scanner high density 256 channel EEG. EEG-fMRI data were obtained on two systems. Of the 39 patients included in these analyses, 34 had seizures during EEG-fMRI. For six patients, the in-scanner EEG data were collected on a modified Quik-Cap 21 channel cap with silver/silver-chloride electrodes (Neuroscan), carbon-fiber cables (in-house), a 125 Hz, analog, low-pass Butterworth filter (in-house), and an EEG recorder (NuAmps, Neuroscan). EEG signals were sampled at 500 Hz with 22-bit resolution. For 27 patients, the in-scanner EEG data were collected using a modified cap with 32 carbon wire electrodes and preamplifier (in-house) (11), and EEG signals were sampled at 1000 Hz with 24-bit resolution (SynAmps2, Neuroscan). Data for one patient were collected on both systems over the course of several visits. Scanner artifact was removed using a combination of SCAN (NeuroScan) software and in-house temporal principal component analysis (PCA) software or adaptive noise cancellation as described previously (4, 5, 11, 12). After artifact removal, EEGs were low-pass filtered at 50 Hz before visual inspection.

Seizures were captured on out-of-scanner high-density EEG for 15 patients (10 with ictal behavior, 11 if including seizures with intermediate performance). Of these patients, 13 (6 with ictal behavior, 9 if including seizures with intermediate performance) also had seizures during EEG-fMRI. For out-of-scanner high-density EEG, caps were placed with 256 Ag/AgCl electrodes with Netstation v 4·2 software (Electrical Geodesics Incorporated (EGI)) and high impedance amplifiers, sampled at 250 samples per second (0·1-100 Hz filter) or 500 samples per second (0·1-250 Hz filter). All electrodes were referenced to Cz for recording. All impedances were kept at or under 40 kΩ prior to the beginning of each recording session. Parameters were otherwise as previously described(13).

**EEG analysis**

EEGs were read by consensus of two reviewers (graduate or medical students) and confirmed by an experienced epileptologist (H.B.) to identify the presence and timing of seizures. Seizure onset was defined as the first spike of the spike-wave discharge, and end was defined as the negative peak of the last slow wave, with times marked within 0·1 s. Although EEG duration of 3 or 4 s has been proposed as a criterion to separate ictal from interictal spike-wave discharges in absence epilepsy (8, 14, 15), there is evidence that even spike-wave discharges lasting less than one second can cause behavioral impairment with careful testing (4, 16-19). We therefore chose to include all spike-wave discharges in our analysis.

Quantitative analyses of EEG signal amplitude (Figures 5 and 6D,E) were not performed on in-scanner data because of its lower quality from MR artifact. Analyses of signal amplitude were performed on the high quality HD-EEG data with MATLAB (MathWorks). EEG runs acquired at 500 Hz were down-sampled to 250 Hz, and HD-EEG signals were re-referenced from Cz to common average reference.

The EEG signal during absence seizures consisted of large amplitude ~3 Hz waves alternating with higher frequency spikes (Figure S1A). To analyze these components separately, we used the 2·5-4 Hz and 10-125 Hz frequency bands to isolate the waves and spikes, respectively (Figure S1B, C; Figure S2). For topographic mapping of seizure amplitude (Figure 5), each seizure and a corresponding baseline period of the same duration and ending two seconds prior to seizure onset were cut out of the continuous EEG acquisition. A notch filter at 60 Hz was used to remove electrical noise. A fast Fourier transform was used to transform seizure and baseline epochs into the frequency domain at each lead. Power for each frequency sample was divided by its corresponding baseline value per electrode, yielding fractional changes in power (seizure/baseline). Mean fractional changes for the 2·5-4 Hz and 10-125 Hz frequency bands were calculated for each lead across seizures, and then plotted in a standard headmap using the MATLAB toolbox FieldTrip (<http://www.ru.nl/fcdonders/fieldtrip>) to assess topographic patterns. Subsets of electrodes were categorized into anterior, middle, and posterior regions (see Figure 5A). To analyze differences in amplitude on a regional basis, we then performed paired t-tests across electrodes in each of the three regions (as well as across all electrodes) for the seizures with impaired versus spared performance on task. Inclusion of seizures for analysis of EEG in relation to behavior used the same criteria described above for the behavioral analysis (i.e., excluding time epochs where interictal performance was clearly affected by sleep or obvious lack of cooperation), and analyzed seizures where behavioral performance was impaired (defined as <25% correct response rate) versus spared (>75% correct response rate). We also repeated the analysis for each task separately (Figures S16, S17) as well as using a 50% behavioral cutoff instead of <25% and >75% criteria (Figure S18). For all of these analyses data were computed across seizures without pooling first within subjects. However we also repeated the analysis for the small subset of subjects who had both spared and impaired seizures, and for that analysis we first pooled data by averaging within subjects before computing means comparing seizures with impaired vs spared behavior within the same subjects (Figure S19).

Next, to determine seizure amplitude changes over time (Figure 6D, E), epochs of peri-ictal and ictal data (-20 s to +60 s relative to seizure onset) were transformed into the frequency domain for each lead in MATLAB. The short-time Fourier transform was computed on non-overlapping time windows (Hamming window width = 1 s) to determine the frequency composition of short sequential data segments over time(20). Time courses were baseline-corrected by dividing all signals by the average interictal signals. For each seizure, the interictal periods were defined as the entire run minus the peri-ictal periods of interest. Mean baseline interictal amplitudes were calculated per lead for the 2·5-4 Hz and 10-125 Hz frequency bands separately. Subsequently, for each lead, the peri-ictal and ictal amplitude averaged across a given frequency band at each time point was divided by the corresponding baseline value per lead and per frequency band to obtain fractional change. To reduce movement artifact, the two outer rows of the 256 channel grid were excluded, and we also excluded from analysis any one second data epoch from the peri-ictal period with amplitude >50% of the average amplitude across leads of the first two seconds in each seizure. Seizures or leads with obvious movement artifacts were also excluded. This led to exclusion of a total of two seizures and to an average of 4-5 leads among the 256 leads per seizure. The time course of fractional changes in the 2·5-4 Hz and 10-125 Hz frequency bands were then averaged across all leads for each time point. Averaging was computed across seizures without first averaging within subjects to maximize the number of seizures included in the analysis. Group averaging across seizures was performed by temporally aligning data to seizure onset and one second epochs were plotted as mean ± SEM. Two-tailed t-tests for corresponding time points were then performed to compare amplitude of seizures with impaired versus spared performance on behavioral tasks as defined above.

**fMRI analysis**

Absence seizures show evolving fMRI signal changes before and after the electrical activity on EEG that do not fit the canonical hemodynamic response function (5, 9, 10). Therefore, our fMRI analyses of seizures used a data-driven approach and included a peri-ictal period from -30 to +58 seconds relative to seizure onset. Because of the large size of both the data set and the number of computations, we carried out these analyses using a combination of a local Linux-based server and the supercomputer cluster at Yale’s Center for High Performance Computing (Xeon E5-2670, 2·6 GHz, 16 cores, 128 GB RAM). In summary, we: 1. used a data set including a large number of seizures to obtain generalizable data-driven network regions as well as region-specific fMRI time courses and hemodynamic response functions for all seizures, and then used these results on the subset of seizures with impaired or spared behavioral performance to perform 2. statistical parametric mapping comparing fMRI signals for impaired versus spared seizures, and 3. fMRI time course analysis of impaired versus spared seizures.

Briefly, fMRI signal over time was first calculated for each voxel. Baseline interictal values were computed per run, by averaging over time excluding peri-ictal and ictal periods. Peri-ictal and ictal periods were then extracted. Percent signal change was calculated at each time point as 100 × (signal – baseline)/baseline . We performed k-means clustering on fMRI peri-ictal and ictal signal percent change time courses to cluster voxels with similar time courses in a large dataset including seizures not used in subsequent analyses. This provided network regions and network-specific hemodynamic response functions, which we then used for statistical parametric mapping (SPM, http://www.fil.ion.ucl.ac.uk/spm) and time course analyses of fMRI changes in impaired vs. spared seizures. fMRI analyses were performed using a combination of in-house software (MATLAB) and SPM.

*Establishing data-driven network regions and mean time courses (Figure 3)*

We used fMRI data from a large set of seizures (n=810) to establish *in general* the regions and time course of fMRI signal changes during absence seizures. For this we included seizures not used in subsequent analyses. That is, in addition to seizures with clearly impaired or spared behavior, we also included seizures with intermediate performance, seizures that occurred during periods of time without a behavioral target, and seizures that occurred during fixation.

Typical fMRI pre-processing steps were first applied to our data. The first ten volumes of each run were discarded to allow for scanner stabilization. Remaining images were spatially realigned to the first image of each functional run and normalized to the template brain “colin27” (single_subj_T1 from SPM) in MNI space in SPM2. Images were then spatially smoothed with an isotropic Gaussian kernel at ten mm FWHM. The standard 1/128 Hz high pass filter from SPM8 was next applied to the data. Analyses were confined to cortical and subcortical gray matter voxels by using a standard gray matter mask from MarsBaR (<http://marsbar.sourceforge.net/>) and adding the midbrain and pons.

Prior to percent change time course and SPM analysis, additional fMRI artifact removal was performed in sequential steps at the level of 1) subjects, 2) runs, and 3) brain volumes (i.e., images of the whole brain acquired every 1·55 s). First, since neuronal sources of BOLD changes typically do not exceed 5%, activity >7% is unlikely to be biological in origin (21). A rater blinded to performance conditions reviewed preliminary percent change signal maps averaged across seizures for individual patients at the 7% threshold level for obvious artifact. Data from four patients were discarded at this step. Next, signal-to-noise ratio was calculated over time for each run [20 × log(mean BOLD signal/standard deviation of BOLD signal)] taking the average over the whole brain. 34 of 237 runs with SNR <30 were discarded at this step.

For the percent change time course analyses, several measures were used to ensure data quality on a volume-by-volume basis. A standard general linear model was used to regress out the effects of motion in six directions as well as the effects of task onset, task offset, and task block durations. Subsequently, instantaneous changes in head position, or framewise displacement (FD), was calculated as the sum of the absolute values of changes in the six parameters for transitional and rotational displacement between volumes (22). The root mean squared (RMS) difference in BOLD signal from volume to volume was also calculated (referred to as DVARS) (22, 23). Volumes exceeding a DVARS threshold of five or an FD threshold of 0·3 were discarded as in previous studies (22, 23) resulting in 15·3% of all volumes discarded from the time course analyses. Seizures occurred at random times relative to image acquisition times during fMRI. Data were therefore interpolated from TR = 1·55 s to 1 s and binned with uniform intervals to align images with standard time epochs relative to seizure onset (defined as t=0).

To further ensure that average fMRI changes were not contaminated by any systematic movement time-locked with seizures, we calculated the mean motion time courses in six directions for the same time intervals as the fMRI time course analysis for all seizures (Figure S3A). This analysis revealed very small average movement in all directions, comparable to that seen in equivalent non-seizure epochs (Figure S3B).

Prior work has shown heterogeneity of the hemodynamic response function (HRF) across brain regions for task in normal subjects (24) as well as during pathological events such as absence seizures (5, 9, 10). To account for this variability in the HRF, we computed voxel-based maps of percent signal change over time for the whole brain. fMRI percent signal changes for seizures were calculated on a voxel-by-voxel basis over time as 100 × (D1-D0)/D0, where D1 was instantaneous signal intensity and D0 was averaged signal intensity over the entire run excluding periods analyzed for seizure. Based on previous studies showing fMRI changes occurring up to 20 seconds before seizure onset and lasting up to 30 seconds after seizure end (5, 9), the time course of fMRI signal was mapped as a peri-ictal and ictal period extending from -30 to +58 seconds relative to seizure onset. For the group analysis, seizures were aligned by onset and averaged. Time course analyses for seizures normalized to an average duration as well as aligned by offset were also performed as in prior work(5) and gave similar results (data not shown).

To further examine BOLD changes associated with ictal activity without assumptions about the shape of the underlying HRF, several data-driven clustering algorithms were applied in MATLAB to the average BOLD signal time courses. To obtain sets of voxels with similar fMRI time courses, k-means clustering was applied (25). We performed 50 replicates per value of k, to validate the selection of the initial cluster centroid locations providing minimal within-cluster distances. There is a tradeoff between finding a small number of functionally related regions and allowing for greater within-cluster heterogeneity versus extracting a greater number of more homogenous clusters. To optimize the number of clusters, we calculated the silhouette value (26), a measure of how well clustered a network is, using k = 2 to 30. For k >3, individual clusters had a significant number of voxels with negative silhouette values (resulting in total cluster silhouette values close to zero), indicating high levels of dissimilarity within those clusters (Figure S4). Therefore we used the k-means clustering algorithm to parcellate the brain voxelwise fMRI time courses into three clusters (k=3) (Figure 3A, B). To determine the mean fMRI time course in each cluster, we aligned seizures by onset time and then averaged the percent change values across voxels within each cluster (Fig 3C).

As additional confirmation we repeated the cluster analysis using hierarchical clustering on a voxel-by-voxel basis (25) and obtained very similar regions to those with k-means clustering (Figures S5, S6). With hierarchical clustering, we again found that using more than three clusters tended to isolate small volumes in areas likely due to noise (Figure S5). The anatomical regions obtained with hierarchical clustering for the first three clusters closely matched those obtained with k-means clustering (Figure S6).

*fMRI statistical parametric mapping of impaired versus spared seizures*

Our next goal was to use this information about fMRI network regions and time courses for the three clusters obtained from the general case (496 seizures) to perform statistical analysis specifically comparing seizures with impaired versus spared behavior. This involved several steps. First we used the three regional fMRI time courses (Figure 3C) to obtain network region-specific hemodynamic response functions (HRFs) for the mean seizure duration. Convolving these HRFs with a boxcar function equal to the mean seizure duration closely reproduced the original time course for each region. Next, to relate seizures of any duration to the fMRI signal we performed linear analysis to find the best fit of boxcar duration to generate fMRI time courses with seizures of different durations. Finally, we used the data-derived HRFs and adjusted boxcar durations from the general data set to perform statistical parametric mapping applied to seizures with impaired versus spared behavior.

i. Region-specific HRFs
To obtain region-specific HRFs, we used a process equivalent to deconvolution (27, 28). For each cluster of voxels derived from the prior analysis (Figure 3), the average BOLD time course (-30 to +58 seconds from seizure onset) was first smoothed with a 25^th^-order polynomial. The smoothed time courses for each cluster were then fit using linear regression against the SPM standard Fourier basis set (time bin = 0·00625 s, window length = 10 s, order = 10) convolved with a boxcar of the average seizure duration. Cluster (region)-specific HRFs were then generated by weighting the Fourier basis set with the resulting regression coefficients (27).

ii. Adjusting boxcar duration for seizure durations

To model the fMRI signals for SPM analysis, it was necessary to obtain predicted fMRI time courses for seizures of any duration. Typically this is done by convolving the HRF by a boxcar function of the event duration, in this case seizures. However, to avoid making assumptions about the relationship between seizure duration and fMRI signal amplitude, our goal was to determine boxcar durations (representing seizures of different durations) which would most closely match the data. To optimize the fit, we divided all seizures with fMRI data from Figure 3 into three duration bins (0 to ≤5 s, >5 to ≤10 s, >10 s). Within each cluster and for each duration bin, the boxcar duration was iteratively adjusted in 0·1 s steps from ten seconds less than the mean seizure duration to ten seconds greater than the mean duration to minimize the absolute difference between the actual fMRI time course and the predicted fMRI time course (obtained by convolution of the derived HRF and the boxcar function). The durations providing the best fit were then taken as the optimized boxcar durations for each duration bin. These optimized boxcar car duration values were averaged across clusters for each duration bin. Least squares regression of seizure duration versus optimized boxcar duration for the three duration bins was used to obtain a linear fit (Figure S7). This relationship was used to adjust the boxcar function duration for each seizure in subsequent SPM analyses. The relationship between adjusted boxcar durations and seizure durations for the three bins was nearly linear and close to unity, so in the end adjusted boxcar durations had minimal effect on the analysis (Figure S6). This linear relationship is in close agreement with another recent study which examined the relationship between spike-wave duration in generalized epilepsy and BOLD fMRI signal amplitude(29).

iii. SPM of impaired versus spared seizures (Figure 4)

Data used in SPM analysis underwent the same pre-processing steps including realignment, normalization, and smoothing described above for the percent change time courses (see *Establishing data-driven network regions and mean time courses*). In addition, the 1/128 Hz filter and artifact removal steps at the level of subjects and runs were identical to those already described. We did not, however, regress out effects of motion or task in advance because these were instead included as regressors in the SPM analysis.

Due to the large size of the data set and number of calculations, even with the use of the Yale supercomputer it was necessary to limit the SPM analysis to peri-ictal and ictal periods extending from -30 s relative to seizure onset to +50 s relative to seizure offset. Our analysis scheme was a group fixed-effect analysis, which was necessary due to the fact that individual subjects could have seizures with impaired performance, spared performance, or both. The variability in number of seizures between subjects, including some subjects who had only one or no seizures within one behavioral category (spared or impaired responses, see Table S1), and the inherent noise in the individual seizure timecourses further reduced the potential power of analysis across subjects. This made individual seizures rather than subjects the most logical unit of analysis, and although fixed-effects provides less inferential power than a mixed-effects model(30-32), this approach allowed us to draw conclusions applicable at least to within our relatively large data sample. The SPM general linear model design matrix consisted of six regressors of motion (3 for translation, 3 for rotation), task onset (5 s boxcar), task offset (5 s boxcar), task block (boxcar of task duration), scan run, as well as regressors for seizures with impaired (<25% correct response rate), spared (>75% correct response rate), and intermediate (25% to 75% correct response rate) behavioral performance. (For Figure S12 the analysis was repeated but with impaired and spared performance redefined as ≤50% or >50% correct response rates, respectively). Seizures were modeled by convolving cluster-specific HRFs with adjusted boxcar durations for each seizure (very similar to seizure duration) as described above. The pre-whitening AR(1) model was applied before estimation, as is standard in SPM8. fMRI changes for impaired or for spared seizures were then determined using a t-contrast in SPM (height threshold t=2·33, extent threshold k=3 voxels, voxel dimensions 4×4×4 mm), masked to the voxels corresponding to the cluster-specific HRF utilized, and corrected for multiple comparisons using small volume correction (SVC)(33, 34) with family-wise error (FWE) threshold p < 0·05 (Figure 4A-F).

To summarize the difference in fMRI amplitude, we next calculated the fMRI percent change for spared and impaired seizures in each cluster, averaged across seizures and compared with a two-sample, two-tailed t-test (Figure 4G). fMRI percent change for each seizure was calculated using the fMRI percent change time course data (see details in *Establishing data-driven network regions and mean time courses* above) by calculating the root-mean-squared percent change from 0 to 40 s after seizure onset. This interval was chosen because the majority of fMRI signal changes occurred within these times (see Figure 6A-C) however very similar results were obtained if calculations were done instead with different intervals such as the entire peri-ictal period, and very similar results were also obtained if peak and trough fMRI changes were analyzed separately instead of analyzing root-mean-squared amplitude (data not shown). fMRI percent change magnitude for each region was thus calculated by taking the square of the percent change value at each time point, taking the mean across time points (from 0 to 40s after seizure onset), and then taking the square root. Analyses for summary histograms and t-tests were again performed across seizures without first pooling results within subjects in order to maximize the ability to study seizure-to-seizure variability in a large data sample.

We repeated the analysis comparing spared and impaired seizures for each task (CPT and RTT) separately (Figure S10, S11). We also repeated the analysis with different choices for behavioral cutoffs other than <25% and >75%, using a single 50% cutoff (Figure S12), or including an intermediate behavioral bin of 25-75% (Figure S13). In addition, we performed a direct contrast of fMRI changes in seizures with impaired vs. spared behavioral performance (Figure S9). Finally, we performed a more stringent analysis on the smaller sample of patients who had both seizures with impaired and spared performance by first contrasting the impaired vs spared seizures within each of these 11 subjects in a first-level fixed-effects analysis, and then entering the results across subjects in a second-level random-effects analysis (Figure S14). Because this last analysis did not show significant differences with our FWE-corrected threshold of p<0·05, we displayed results using an uncorrected threshold of p<0.01 to show the trend towards larger amplitude in seizures with behavioral impairment in this subgroup. In this case for the summary histograms (Figure S14D) unlike the other fMRI histogram calculations above, we first calculated mean values within each subject separately for all seizures with impaired vs spared behavioral responses, and then used the resulting 2 values per subject to perform paired t-tests.

*fMRI time course analysis of impaired versus spared seizures*

Data for fMRI percent change time courses first underwent all pre-processing and artifact removal steps described above (see *Establishing data-driven network regions and mean time courses*). For each seizure with impaired or spared behavioral performance, we then calculated fMRI percent change time courses within each cluster spanning the time period from -30 to +58 from seizure onset. Mean and standard error time courses were then calculated across the set of spared and set of impaired seizures separately (Figure 6A-C). The time points with the most significant differences between impaired and spared seizures were identified using a two-tailed t-test as described previously (9, 35).

**Figure S1**

**
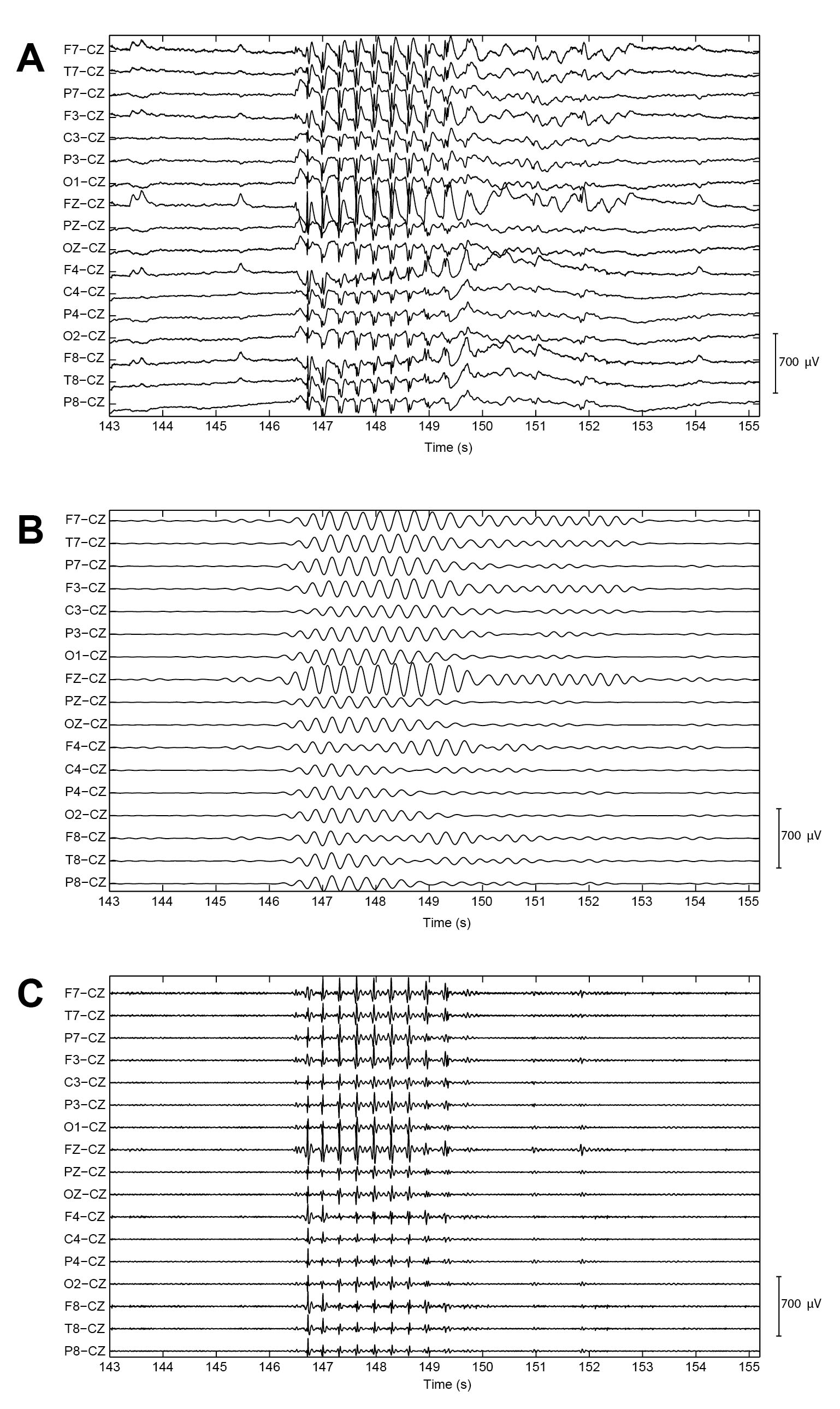
**

**Figure S1** EEG from typical absence seizure consists of low-frequency ~3Hz waves and high-frequency spikes. **A.** Example of EEG changes during an absence seizure showing typical spike-wave discharge. Recording performed out-of-scanner with high-density EEG is shown here with only limited number of channels and bipolar montage for ease of viewing. **B.** Application of 2·5 – 4 Hz bandpass filter to seizure from (A) reveals rhythmic slow waves. **C.** Application of 10 – 125 Hz bandpass filter to seizure from (A) reveals high-frequency spike components.

**Figure S2**


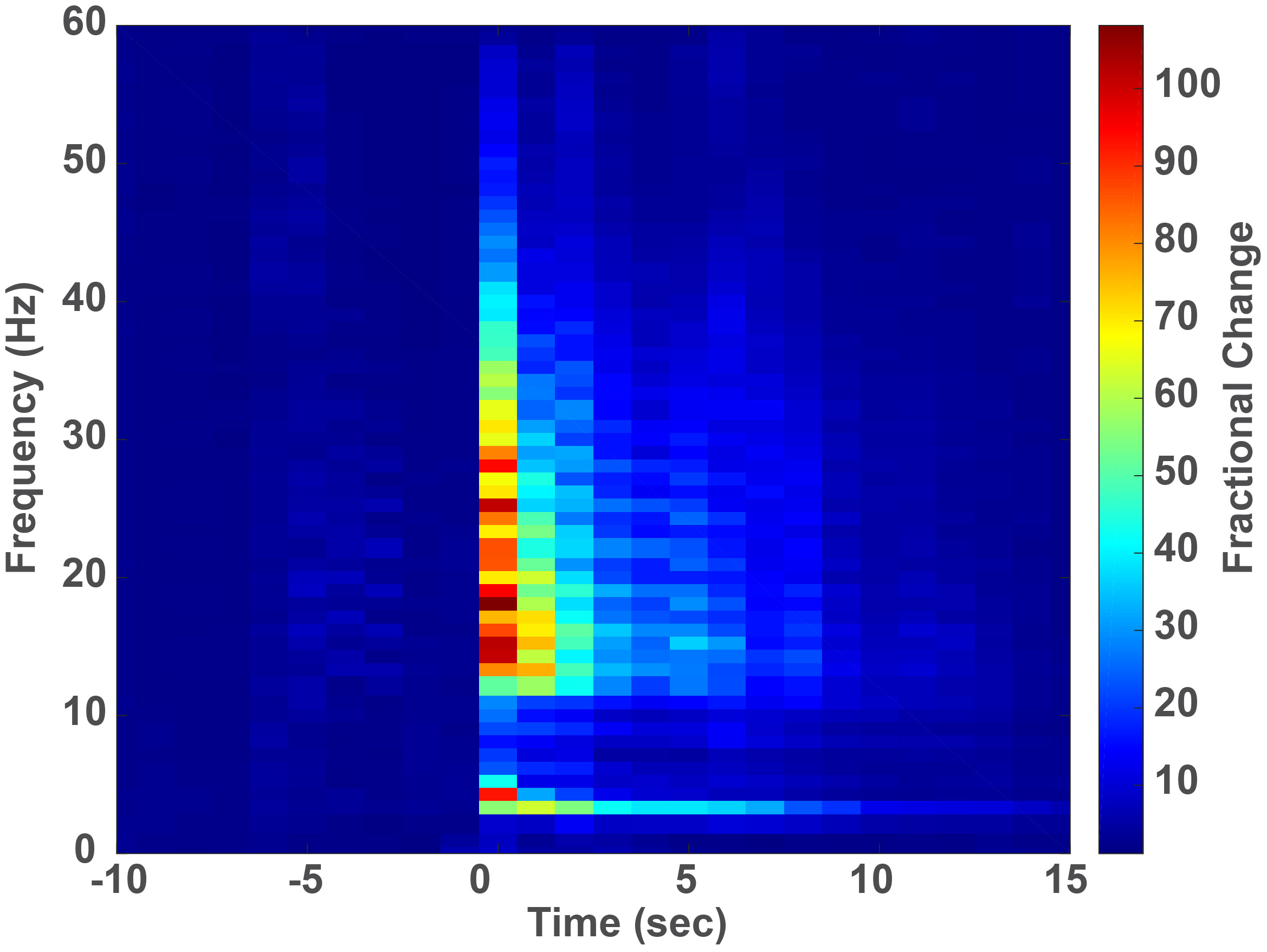


**Figure S2** Average time–frequency dynamics of spike-wave discharges recorded with high-density EEG. Analysis was performed using a short-time Fourier transform with one second, non-overlapping time windows covering the peri-ictal and ictal periods of -20 to 60 s relative to seizure onset and the frequency range of 0 to 125 Hz. For display purposes a more limited range of time and frequency is shown here (only very low amplitude changes were seen outside this range). All ictal periods were temporally aligned to seizure onset. The power is plotted as fractional change by dividing the power at each time point by the mean interictal power at that frequency. Results were averaged across all leads for each seizure, then across seizures. The dominant frequency components of the EEG signal were at 3–4 Hz and >10 Hz (mainly 10-50 Hz) corresponding to the wave and spike components of absence seizures, respectively. n = 138 seizures in 12 patients.

**Figure S3**


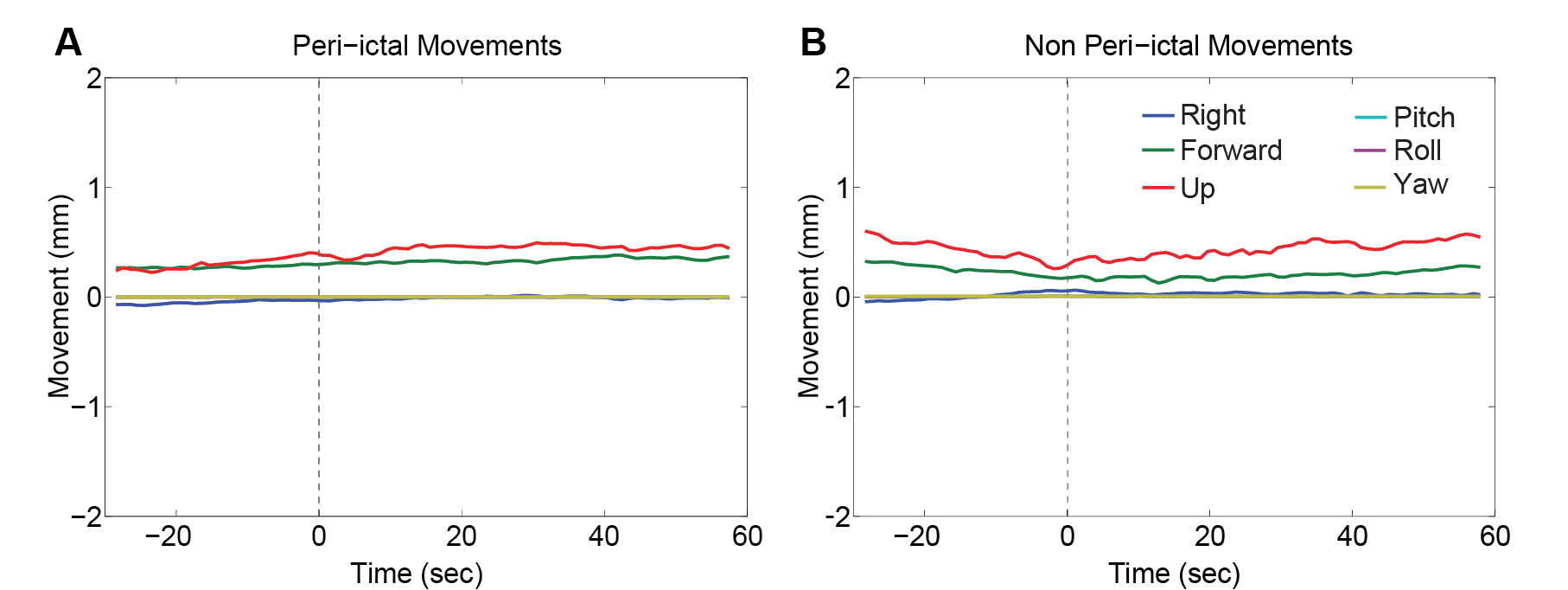


**Figure S3** Average motion plots show no systematic movement time-locked to seizures. **A.** Mean movement time courses in six directions. These include three translational movements of right, forward, and up along the x, y and z axes, as well as three rotational movements around the x, y, and z axes corresponding respectively to pitch, roll, and yaw. These movements are obtained from the SPM realignment step, using the same time epochs as the seizure-related fMRI changes in Figure 3. Like the fMRI data in Figure 3, the movement data here were analyzed between -30s and +58s relative to seizure onset (t = 0). This analysis revealed very small average movement in all directions, with no perceptible changes synchronized with seizures. **B.** For comparison, mean movement time courses were calculated using equivalent epochs selected at random from non-seizure periods in the same run as each seizure. The small average movement observed was comparable to that seen in seizure epochs (**A**). Note that the values for pitch and roll were very small and are covered in these plots by the traces for yaw.

**Figure S4**

**
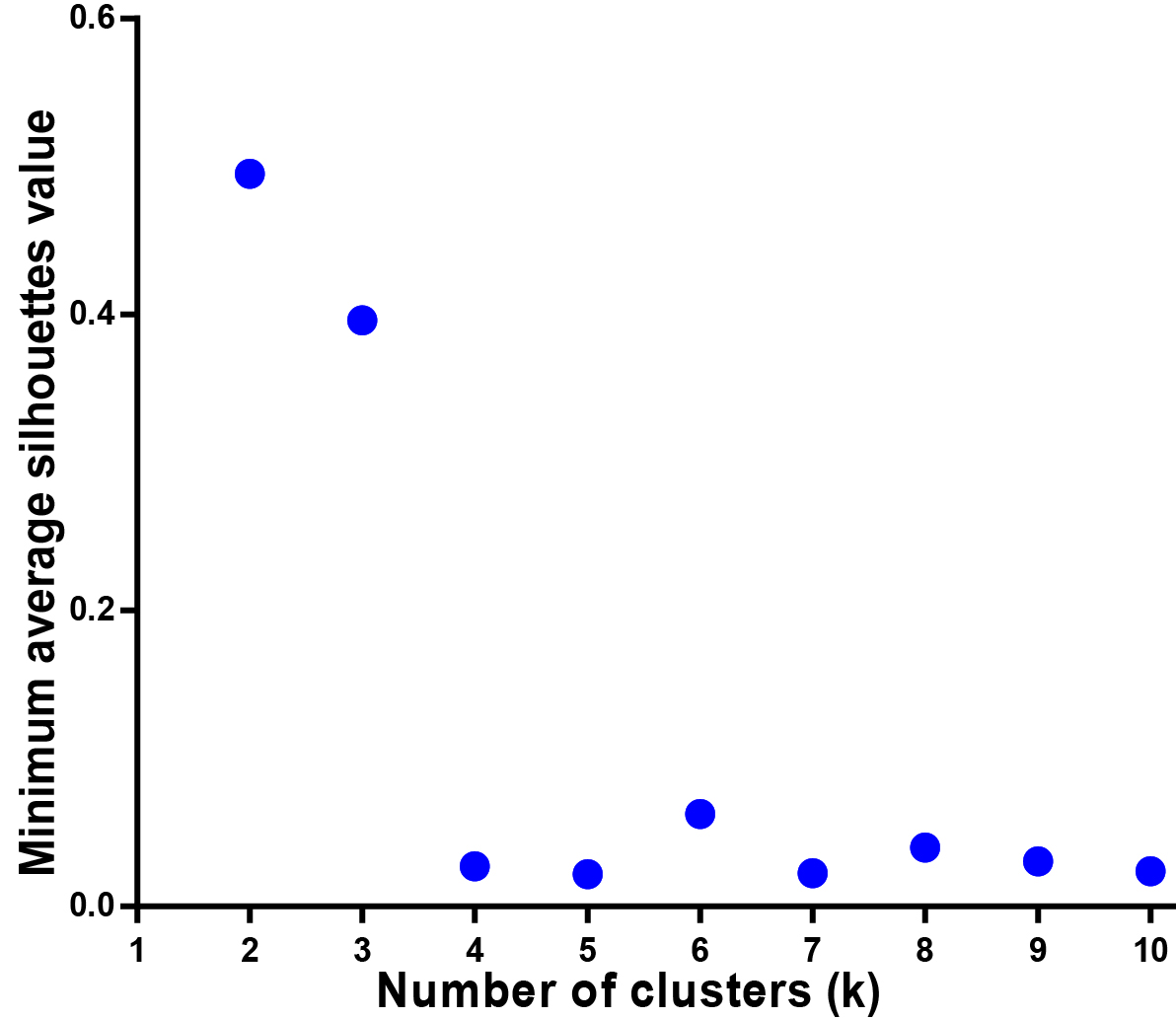
**

**Figure S4** Effect of number of clusters on within-cluster similarity for fMRI time course data. Average silhouette values (a measure of within-cluster similarity) were calculated for each cluster using different total numbers of clusters (k). The lowest (minimum) average silhouette value is shown here for each choice of k from two through ten, demonstrating that for k > 3, clusters emerge with minimum average silhouette values close to zero indicating poor within-cluster similarity. Continuing to increase k up to 30 yielded similar results (not shown).

**Figure S5**

**
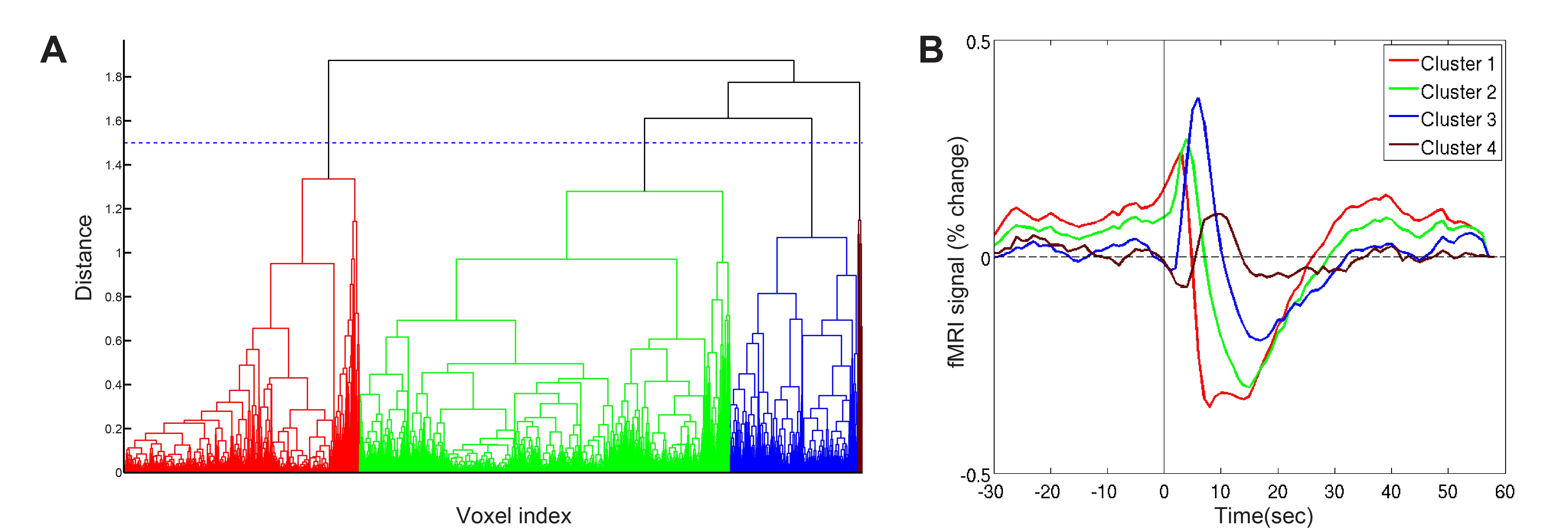
**

**Figure S5** Hierarchical clustering of fMRI time courses shows similar results to k-mean clustering. **A.** Dendrogram of hierarchically clustered voxels (horizontal axis) based on distance (vertical axis), where distance = 1 – correlation between voxels. Horizontal dotted line indicates cut of the dendrogram at 4 clusters, displayed in four corresponding colors in **A** and **B**. **B.** Time course of mean fMRI changes in each cluster (0 = seizure onset). Three clusters (Cluster 1, 2 and 3) had very similar time courses to those seen with k-means clustering for the default mode network, task-positive network, and primary sensorimotor-thalamic network (compare to Figure 3C). The fourth cluster (Cluster 4) was a relatively small (143 voxels) region mainly in the cerebellum with low signal amplitude. Data are from same patients and seizures as in Figure 3.

**Figure S6**

**
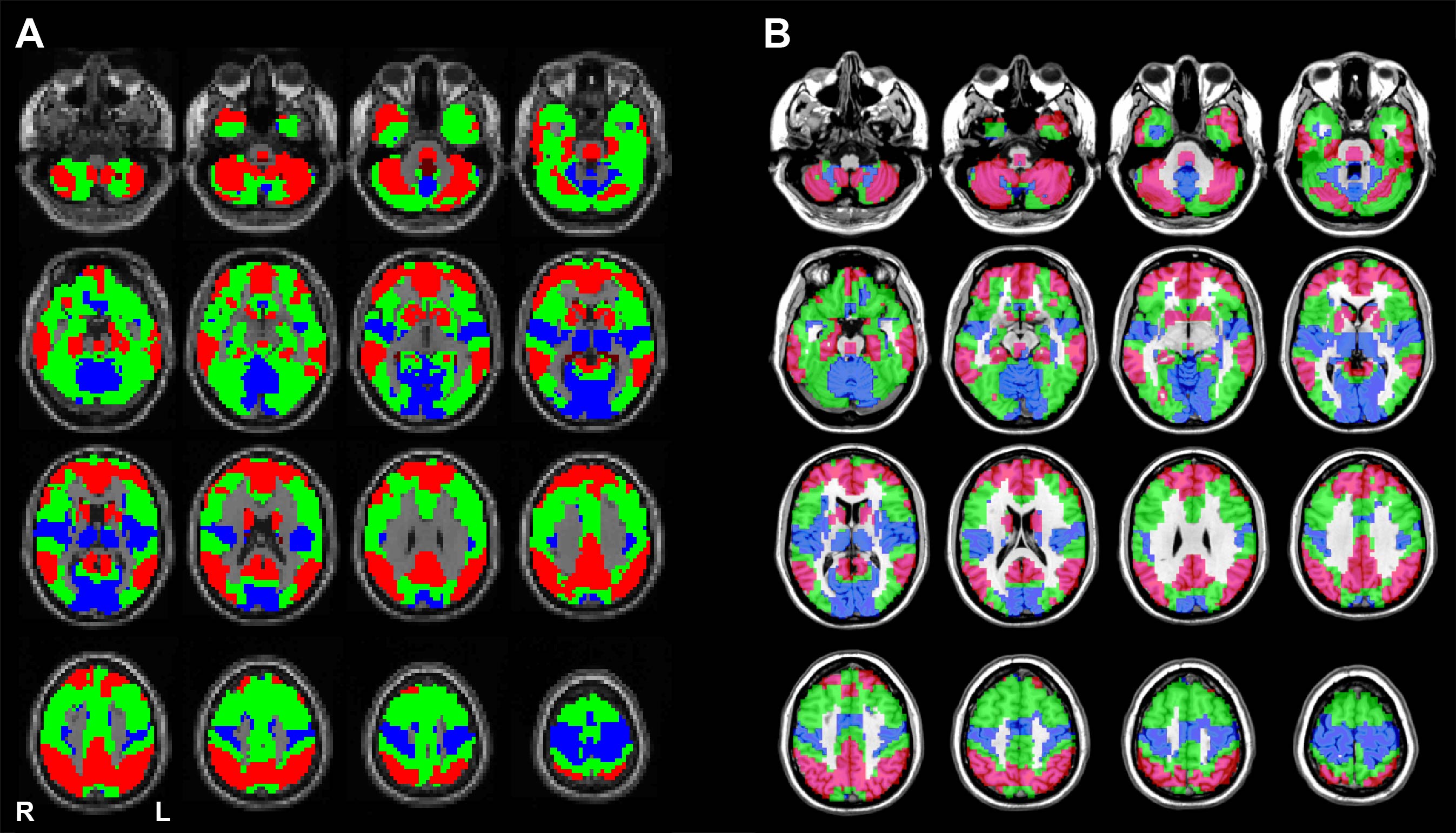
**

**Figure S6** Hierarchical clustering yields similar regions to k-means clustering. **A.** Hierarchical clustering results from Figure S4 rendered on axial brain slices showing parcellation of fMRI activity into distinct networks during seizures. Similar to k-means clustering, these networks encompass the default mode network (red, Cluster 1 from Figure S4), task-positive network (green, Cluster 2), and primary sensorimotor-thalamic network (blue, Cluster 3). An additional small cluster (brown, Cluster 4) includes scattered voxels in the lower cerebellum and adjacent to the lateral ventricles. **B.** Results of k-means clustering with k = 3 for comparison shows similar regions to hierarchical clustering. Data are from same patients and seizures as in Figure 3.

**Figure S7

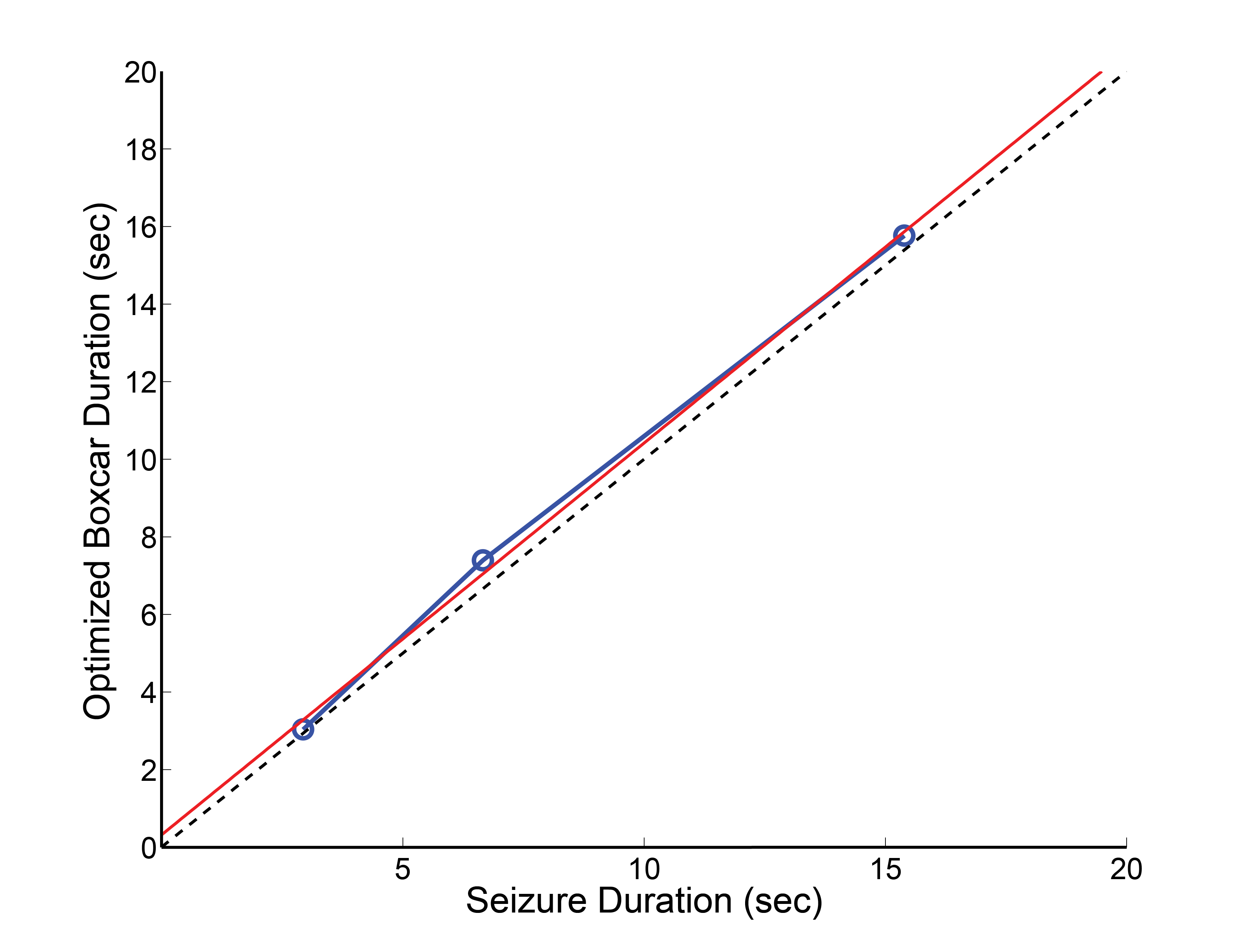
**

**Figure S7** Data-based optimized boxcar durations closely match mean seizure durations for three duration bins. All seizures with fMRI data from Figure 3 were divided into three duration bins (0 to ≤5 s, >5 to ≤10 s, >10 s) and boxcar durations were adjusted for optimal fit to the data within each bin. Optimized boxcar durations are plotted here against mean seizure duration in each bin showing a linear relationship with close to unity slope (represented by dashed line). Linear regression fit is indicated by the red line. Data are from the same seizures and patients as in Figure 3.

**Figure S8**

**
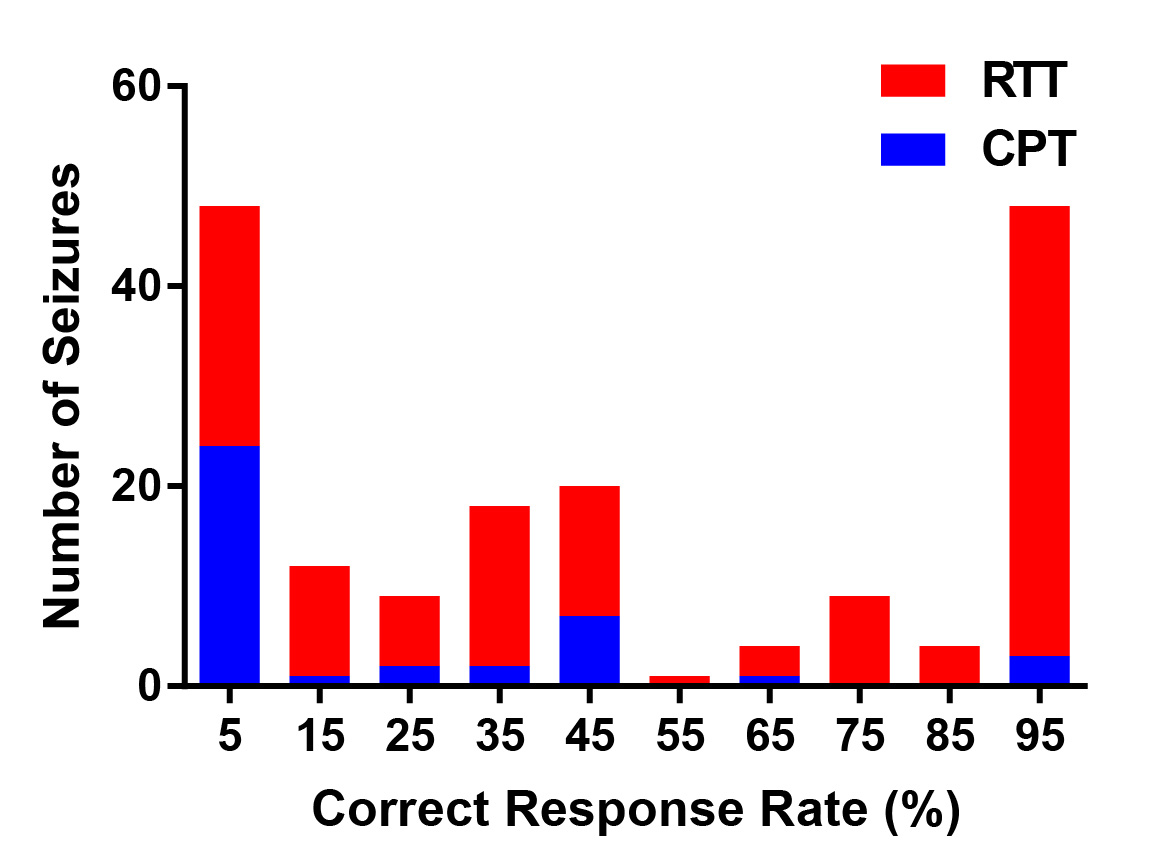
**

**Figure S8** Distribution of task performance (% targets with correct response) for individual seizures with the two tasks, excluding seizures with only one target presentation. Bimodal distribution of behavioral responses is again noted, though proportionally more seizures demonstrate behavior with intermediate performance compared to Figure 2B. Continuous performance task (CPT): 40 seizures in 16 patients. Repetitive tapping task (RTT): 133 seizures in 30 patients.

**Figure S9**

**
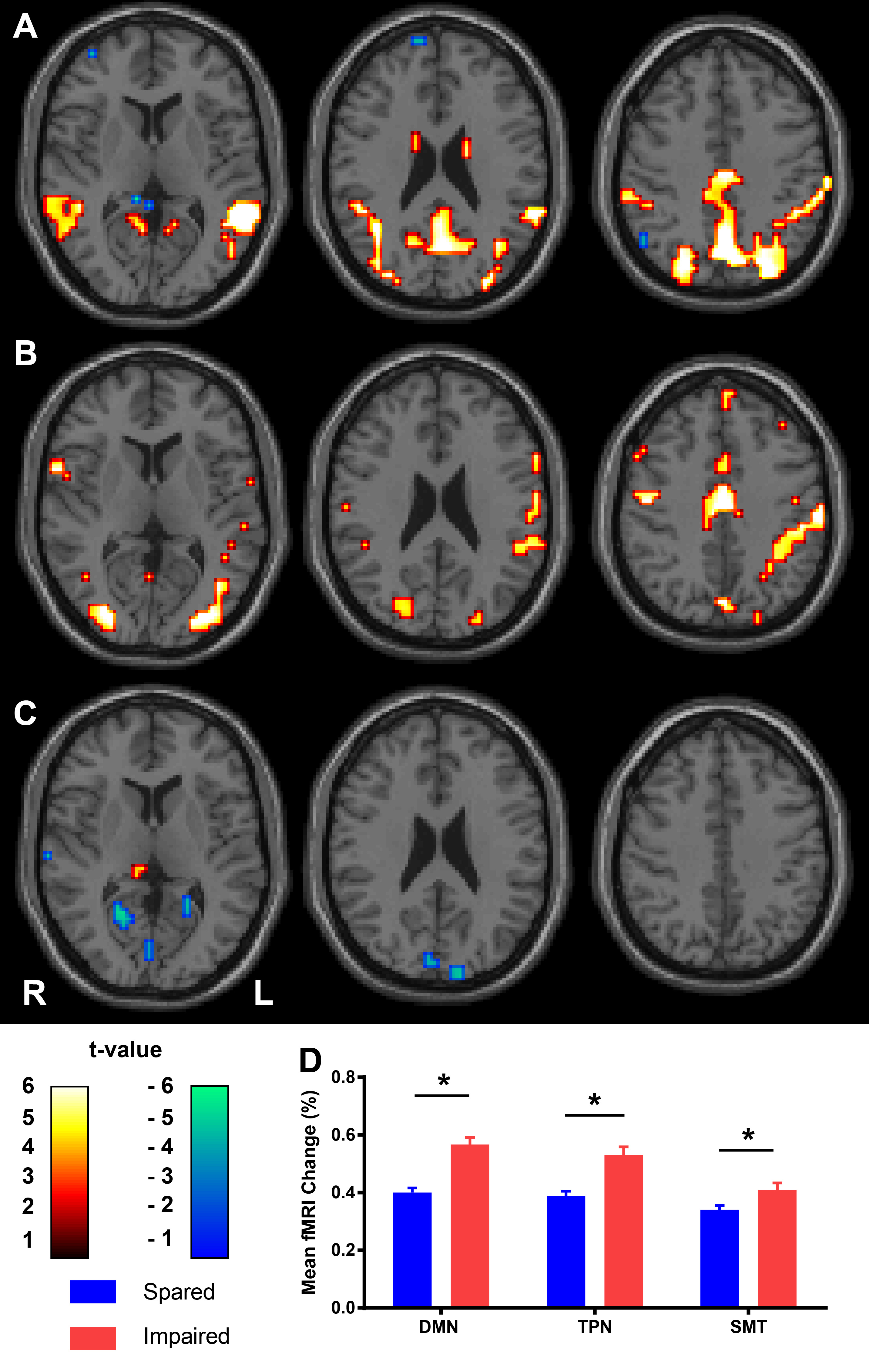
**

**Figure S9** T-maps directly contrasting seizures associated with impaired vs. spared performance using same data as in Figure 4. (A-C) Axial brain t-maps for the default-mode network (A, DMN), task-positive network (B, TPN), and primary sensorimotor-thalamic network (C, SMT). Hot colors indicate regions with significantly greater fMRI changes in impaired than spared seizures, while cool colors indicate the opposite. Significance threshold was p < 0·05 with family-wise error correction for multiple comparisons. Overall larger amplitude fMRI changes for impaired seizures are seen in all three networks (D). Although in (C) seizures with spared performance show more significant changes in some regions (e.g. occipital cortex) other regions show greater changes with impaired seizures (including slices not shown) so that SMT still shows greater overall fMRI changes in seizures with impaired performance (D). As in Figure 4, mean differences (95% CI): DMN 0·17% (0·11 to 0·23%); TPN 0·14% (0·08 to 0·21%); SMT 0·07% (0·01 to 0·12%). Two-tailed two-sample t-tests for seizures with impaired vs seizures with spared task performance, DMN p < 0·0001; TPN p < 0·0001; SMT p< 0·05. n = 93 spared seizures in 17 patients, and 112 impaired seizures in 22 patients.

**Figure S10**

**
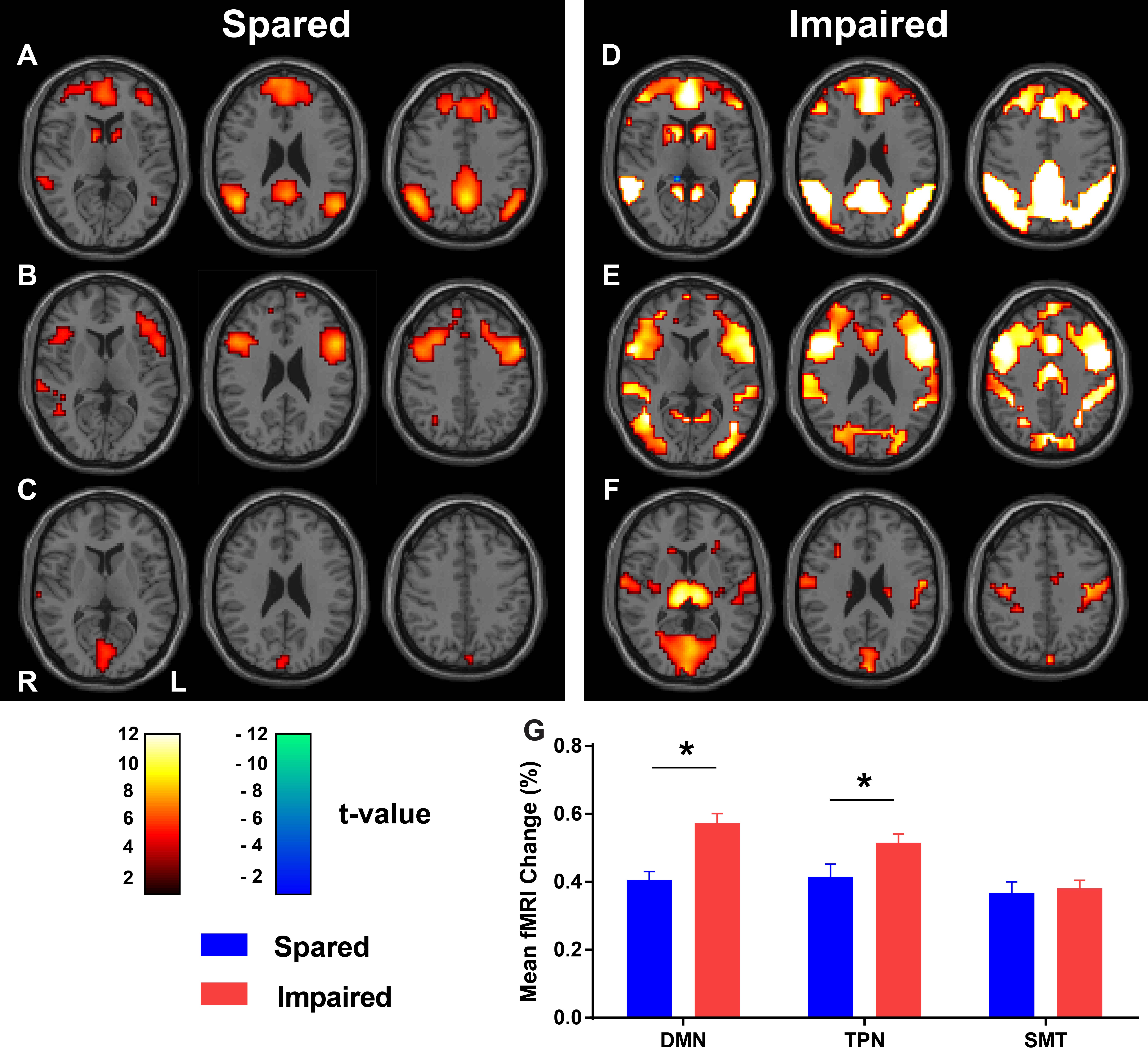
**

**Figure S10** Larger fMRI signals during seizures with impaired performance on the CPT task. Confirmation of results from Figure 4 with single task subgroup analysis. **A-C.** Axial brain t-maps with fMRI signals for seizures with spared performance in the default mode network (**A**, DMN), task positive network (**B**, TPN), and primary sensorimotor-thalamic network (**C**, SMT). **D-F**. Corresponding t-maps for seizures with impaired performance. More significant fMRI changes were seen in widespread regions for seizures associated with impaired performance. **G.** Mean percent fMRI signal change across voxels in each network for seizures with impaired vs. spared task performance. Mean differences (95% CI): DMN 0·17% (0·07 to 0·27%); TPN 0·10% (0·003 to 0·20%); SMT 0·01% (-0·07 to 0·10). Two-tailed two-sample t-tests for seizures with impaired vs spared task performance, DMN p < 0·01; TPN p < 0·05; SMT NS. Analysis was performed using statistical parametric mapping with hemodynamic response functions (HRFs) that are specific for each network. Hot colors in (A-F) indicate brain regions that show significant fMRI changes in the same direction as the network-specific HRFs. Cool colors indicate changes in the opposite direction. Significance threshold was p < 0·05 with family-wise error correction for multiple comparisons. n = 25 spared seizures in 9 patients, and 72 impaired seizures in 16 patients.

**Figure S11


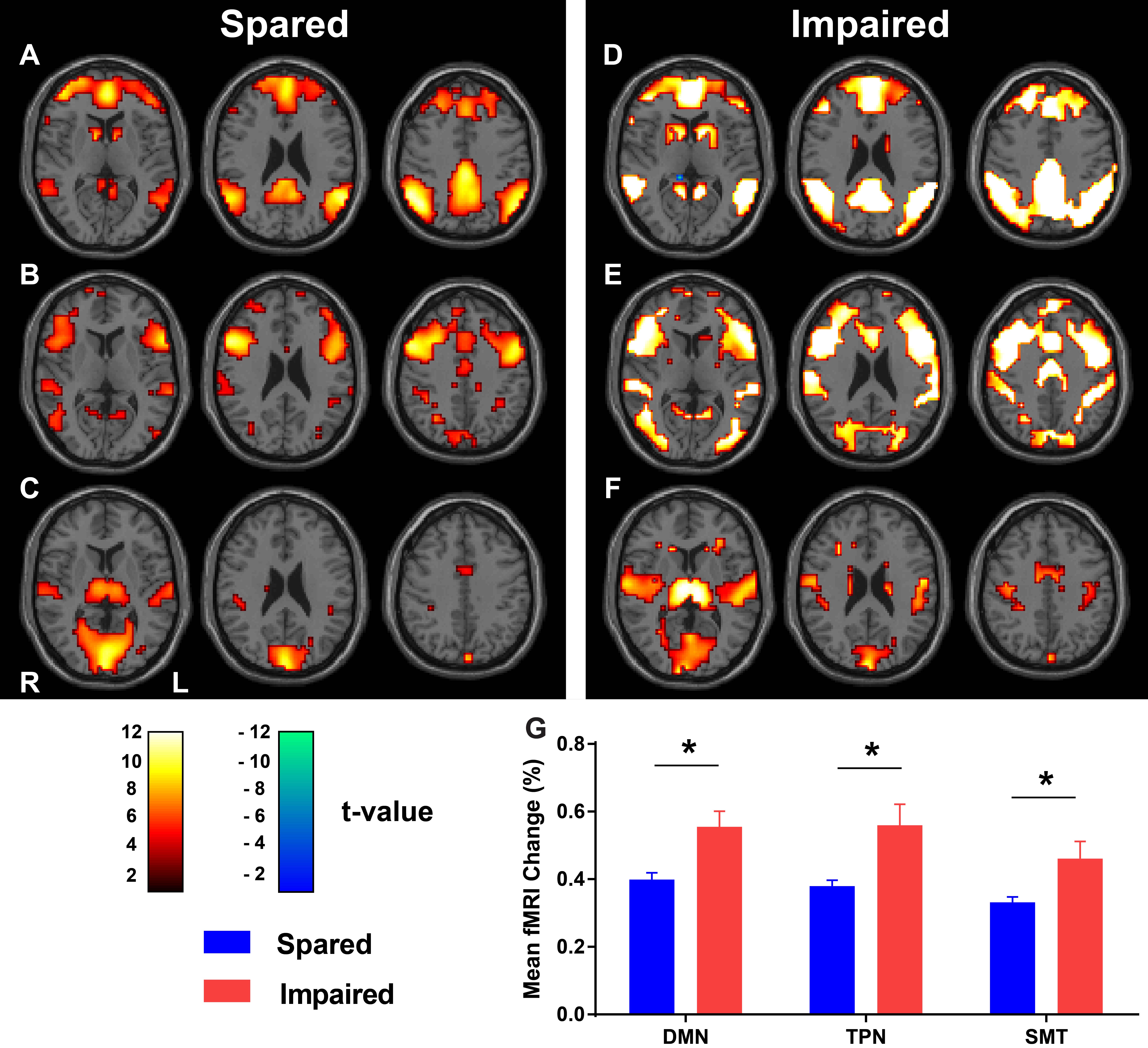
**

**Figure S11** Larger fMRI signals during seizures with impaired performance on the RTT task. Confirmation of results from Figure 4 with single task subgroup analysis. **A-C.** Axial brain t-maps with fMRI signals for seizures with spared performance in the default mode network (**A**, DMN), task positive network (**B**, TPN), and primary sensorimotor-thalamic network (**C**, SMT). **D-F**. Corresponding t-maps for seizures with impaired performance. Greater fMRI changes were seen in each of the three networks in widespread regions for seizures associated with impaired performance. **G.** Mean percent fMRI signal change across voxels in each network were significantly greater in seizures with impaired task performance. Mean differences (95% CI): DMN 0·16% (0·07 to 0·24%); TPN 0·18% (0·08 to 0·28%); SMT 0·13% (0·04 to 0·22%). Two-tailed two-sample t-tests for seizures with impaired vs seizures with spared task performance, DMN p < 0·001; TPN p < 0·001; SMT p < 0·005. Analysis was performed using statistical parametric mapping with hemodynamic response functions (HRFs) that are specific for each network. Hot colors in (A-F) indicate brain regions that show significant fMRI changes in the same direction as the network-specific HRFs. Cool colors indicate changes in the opposite direction. Significance threshold was p < 0·05 with family-wise error correction for multiple comparisons. n = 68 spared seizures in 15 patients, and 40 impaired seizures in 14 patients.

**Figure S12**

**
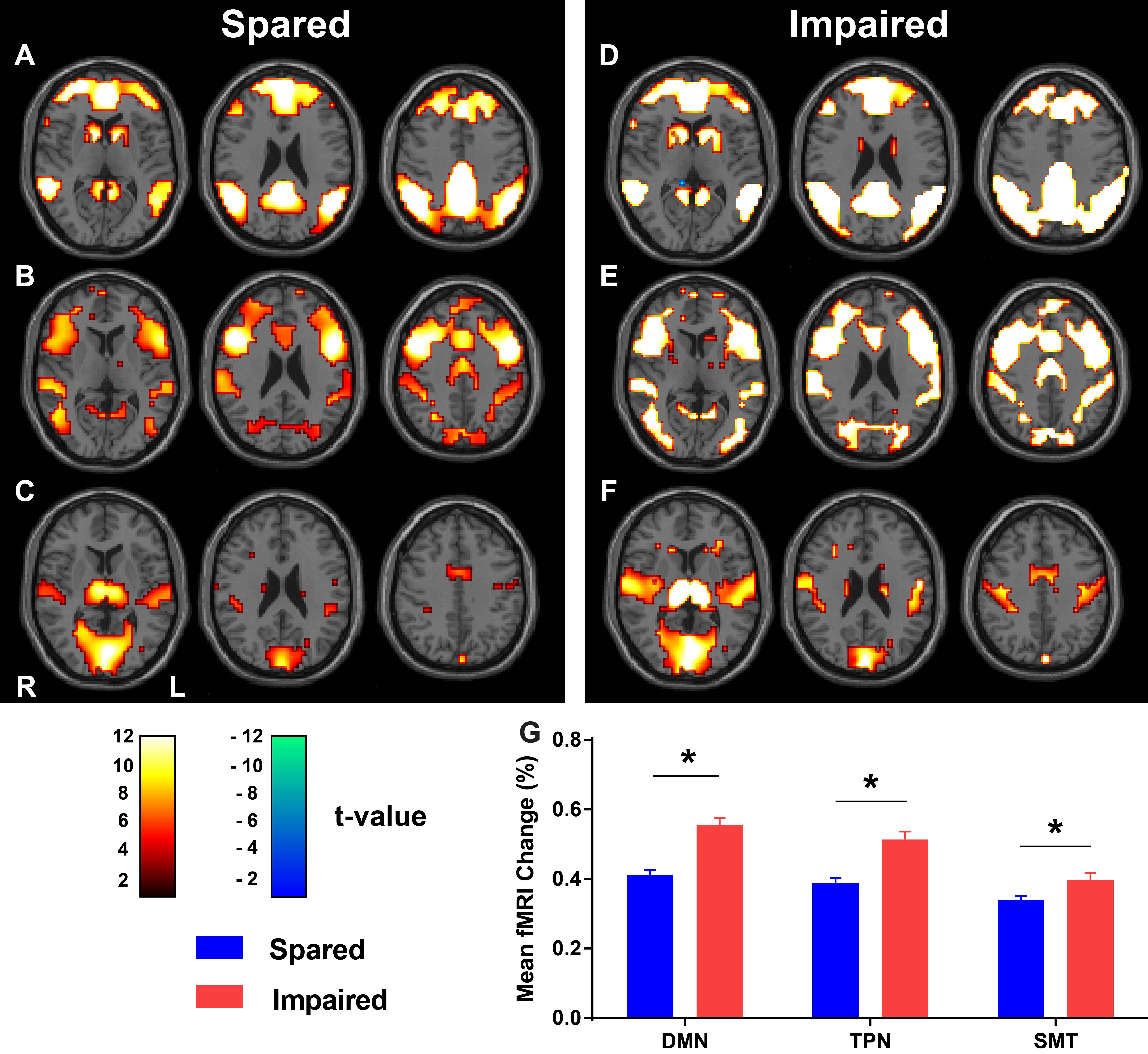
**

**Figure S12** Confirmation of Figure 4 using broader definitions of behaviorally impaired and spared seizures. As in Figure 4, data are from both the CPT and RTT tasks, but here we defined ‘Impaired’ seizures as those with ≤50% correct response rates (instead of <25%), and ‘Spared’ seizures as those with >50% correct response rates (instead of >75%) to behavioral targets. **A-C.** Axial brain t-maps with fMRI signals for seizures with spared performance in the default mode network (A, DMN), task positive network (B, TPN), and primary sensorimotor-thalamic network (C, SMT). **D-F.** Corresponding t-maps for seizures with impaired performance. Greater fMRI changes were seen in each of the three networks in widespread regions for seizures associated with impaired performance. Analysis was performed using statistical parametric mapping with hemodynamic response functions (HRFs) that are specific for each network. Hot colors indicate brain regions that show significant fMRI changes in the same direction as the network-specific HRFs. Cool colors indicate changes in the opposite direction. Significance threshold was p < 0·05 with family-wise error correction for multiple comparisons. **G.** Mean percent fMRI signal change across voxels in each network were significantly greater in seizures with impaired task performance. Mean differences (95% CI): DMN 0·14% (0·09 to 0·18%); TPN 0·13% (0·08 to 0·18%); SMT 0·07% (0·02 to 0·11%). Two-tailed two-sample t-tests for seizures with impaired vs spared task performance, DMN p < 0·0001; TPN p < 0·0001; SMT p< 0·005. n = 117 spared seizures in 18 patients, and 151 impaired seizures in 26 patients.

**Figure S13**

**
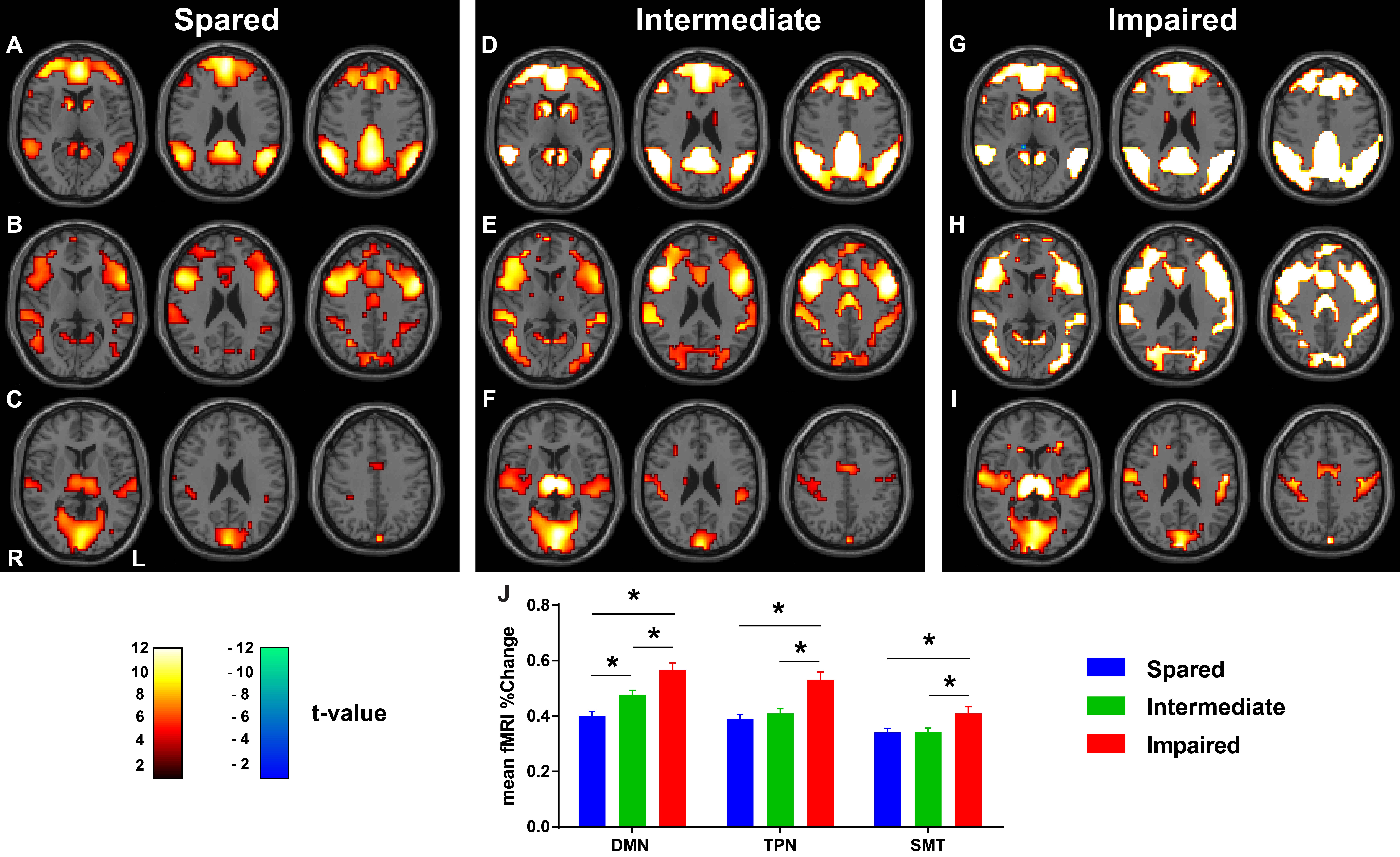
**

**Figure S13** Confirmation of Figure 4 including a separate classification for seizures with intermediate behavioral performance. As in Figure 4, we defined ‘Impaired’ seizures as those with <25% correct response rates to behavioral targets (CPT or RTT), and ‘Spared’ seizures as those with >75% correct response rates; but added a new category of ‘Intermediate’ seizures with ≥ 25% or ≤ 75% correct response rates. **A-C.** Axial brain t-maps with fMRI signals for seizures with spared performance in the default mode network (A, DMN), task positive network (B, TPN), and primary sensorimotor-thalamic network (C, SMT). **D-F.** Corresponding t-maps for seizures with intermediate performance. **G-I.** Corresponding t-maps for seizures with impaired performance. Stepwise increases in fMRI changes were seen in each of the three networks in widespread regions for seizures with progressively worse performance from spared, to intermediate, to impaired. **J.** Mean percent fMRI signal change across voxels in each network for seizures with spared, intermediate or impaired task performance. *p < 0·05 for most comparisons by two-tailed two-sample t-test. Analysis was performed using statistical parametric mapping with hemodynamic response functions (HRFs) that are specific for each network. Hot colors in (A-I) indicate brain regions that show significant fMRI changes in the same direction as the network-specific HRFs. Cool colors indicate changes in the opposite direction. Significance threshold was p < 0·05 with family-wise error correction for multiple comparisons. n = 93 spared seizures in 17 patients, 106 intermediate seizures in 18 patients, and 112 impaired seizures in 22 patients.

**Figure S14**

**
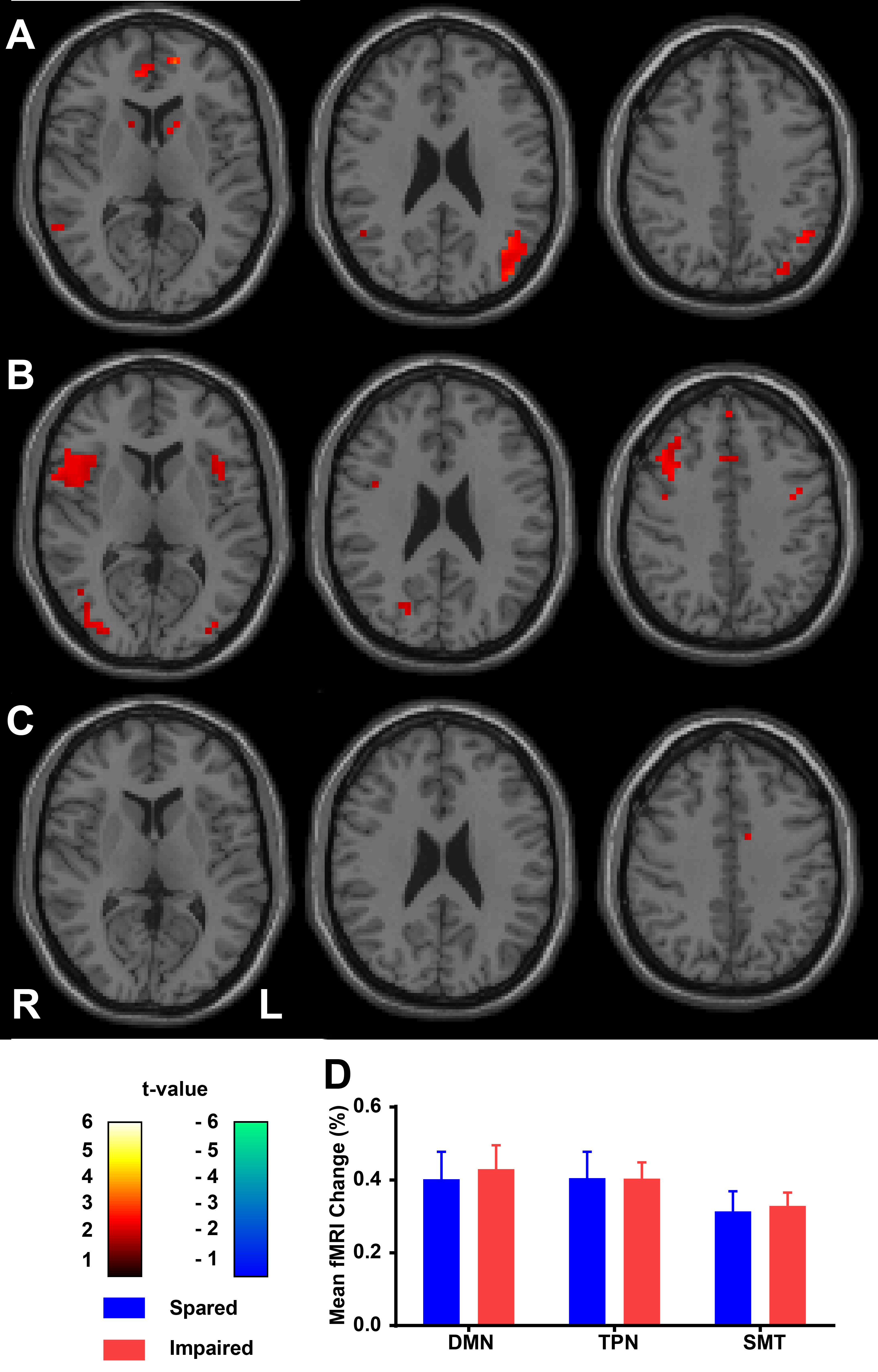
**

**Figure S14** Subgroup analysis of both spared and impaired seizures within the same patients. Due to low sample size (n=11 patients) second-level random-effects analysis yielded no significant differences with family-wise error corrected threshold p < 0·05 (not shown). **A-C.** With uncorrected threshold p < 0·1, axial t-maps contrasting seizures with impaired vs. spared performance within subjects showed larger changes in impaired seizures, although this did not reach statistical significance. **A.** Default mode network (DMN); **B.** Task positive network (TPN); **C.** Primary sensorimotor-thalamic network (SMT). Hot colors indicate larger t-values for seizures with impaired performance, and cool colors the opposite. **D.** Mean percent fMRI signal change across voxels in each network tended to be somewhat larger for seizures with impaired task performance, but was not significant. Mean differences (95% CI): DMN 0·03% (-0·18 to 0·24), p = 0·5; TPN -0·001% (-0·18 to 0·18), p = 0·6; SMT 0·02% (-0·12 to 0·15), p = 0·1, two-tailed two-sample t-test. n = 11 patients (with 81 spared seizures and 78 impaired seizures).

**Figure S15**

**

**

**Figure S15** EEG examples show larger amplitude of seizures with impaired behavioral performance. **A.** Seizure with impaired performance on CPT task. **B.** Seizure with spared performance on CPT task. **C.** Seizure with impaired performance on RTT task. **D.** Seizure with spared performance on RTT task. EEG examples are from out-of-scanner high-density EEG, shown here with only limited number of channels and bipolar montage for ease of viewing. Target letters for behavioral tasks are indicated by vertical lines. For the CPT task (A, B), the targets consisted of the letter “X” presented in a stream of other letters (not shown) appearing once per second. For the RTT task (C, D), the targets consisted of any letter presented once per second. Responses to the targets by button presses are indicated by heavy black lines with duration equal to button press duration.

**Figure S16**

**
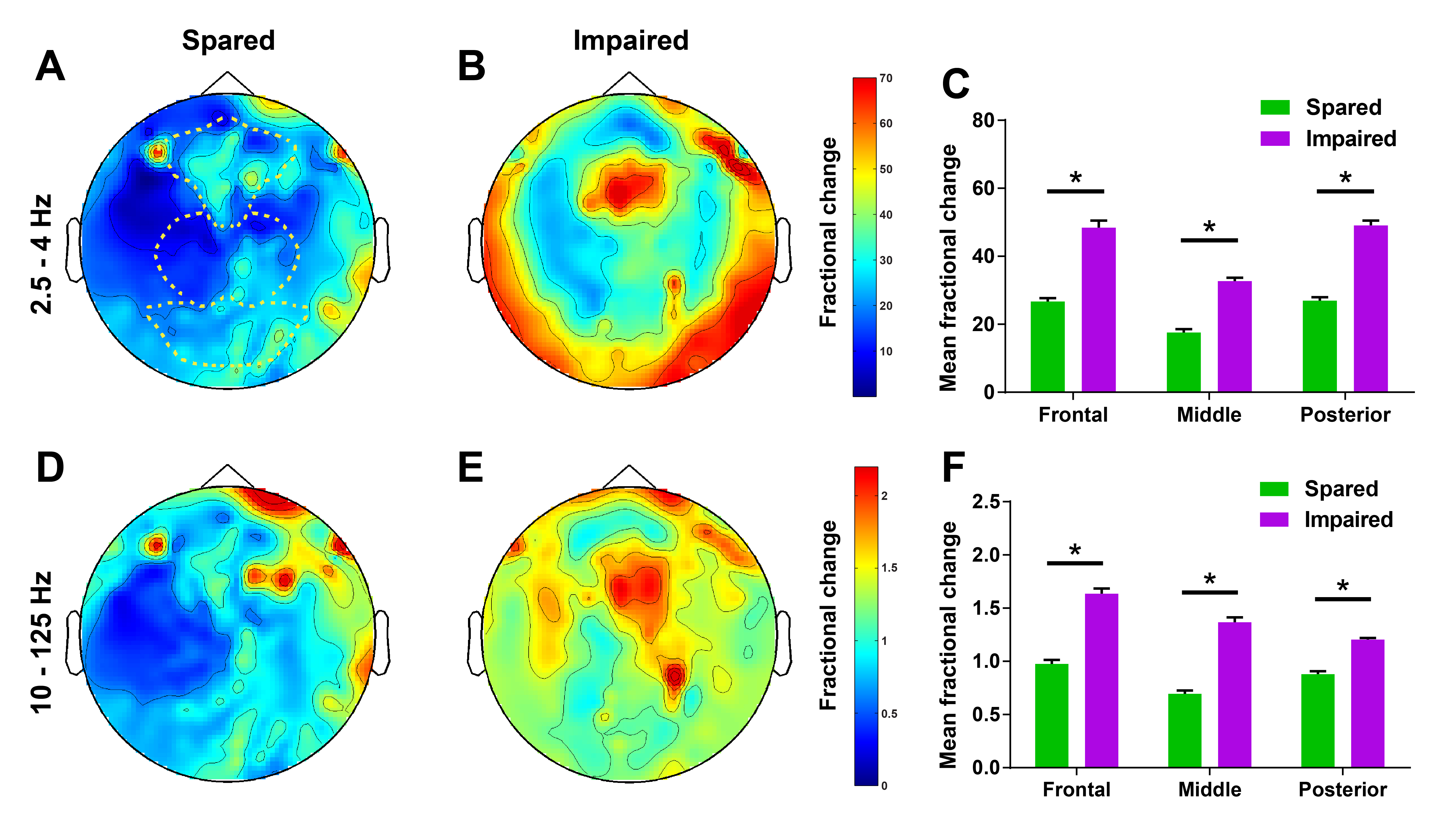
**

**Figure S16** Greater EEG amplitude in widespread brain regions during seizures with impaired performance on the CPT task. Confirmation of results from Figure 5 with single task subgroup analysis. **A-B.** Head maps of 256 channel high-density EEG power in the 2·5-4 Hz frequency range, representing the wave components of spike-wave discharges. Seizures with impaired task performance (B) demonstrate greater power in widespread regions compared to seizures with spared task performance (A). **C.** Greater 2·5-4 Hz power for impaired seizures was independent of electrode position (differences between means in fractional EEG power (95% CI): Frontal leads 21·7 (17·2 to 26·2); Middle leads 15·1 (12·3 to 17·9); Posterior leads 22·1 (18·8 to 25·4), p < 0·001 for all comparisons, paired t-test across electrodes in each region). **D-E.** Maps of EEG power in the 10-125 Hz frequency range (spike components of spike-wave discharges) for spared (D) and impaired (E) seizures. **F.** Greater 10-125 Hz EEG power for impaired seizures was independent of electrode position (differences between means in fractional EEG power (95% CI): Frontal leads 0·66 (0·54 to 0·78); Middle leads 0·67 (0·57 to 0·78); Posterior leads 0·32 (0·26 to 0·39), p < 0·001 for all comparisons, paired t-test across electrodes in each region). Color scale bars represent EEG power during seizures divided by baseline power prior to seizures (fractional power). Regions used for analysis in (C) and (F) (Frontal, Middle, Posterior) are shown by dashed lines in (A). Error bars are SEM. n = 6 spared seizures in 2 patients, and 10 impaired seizures in 5 patients.

**Figure S17**

**
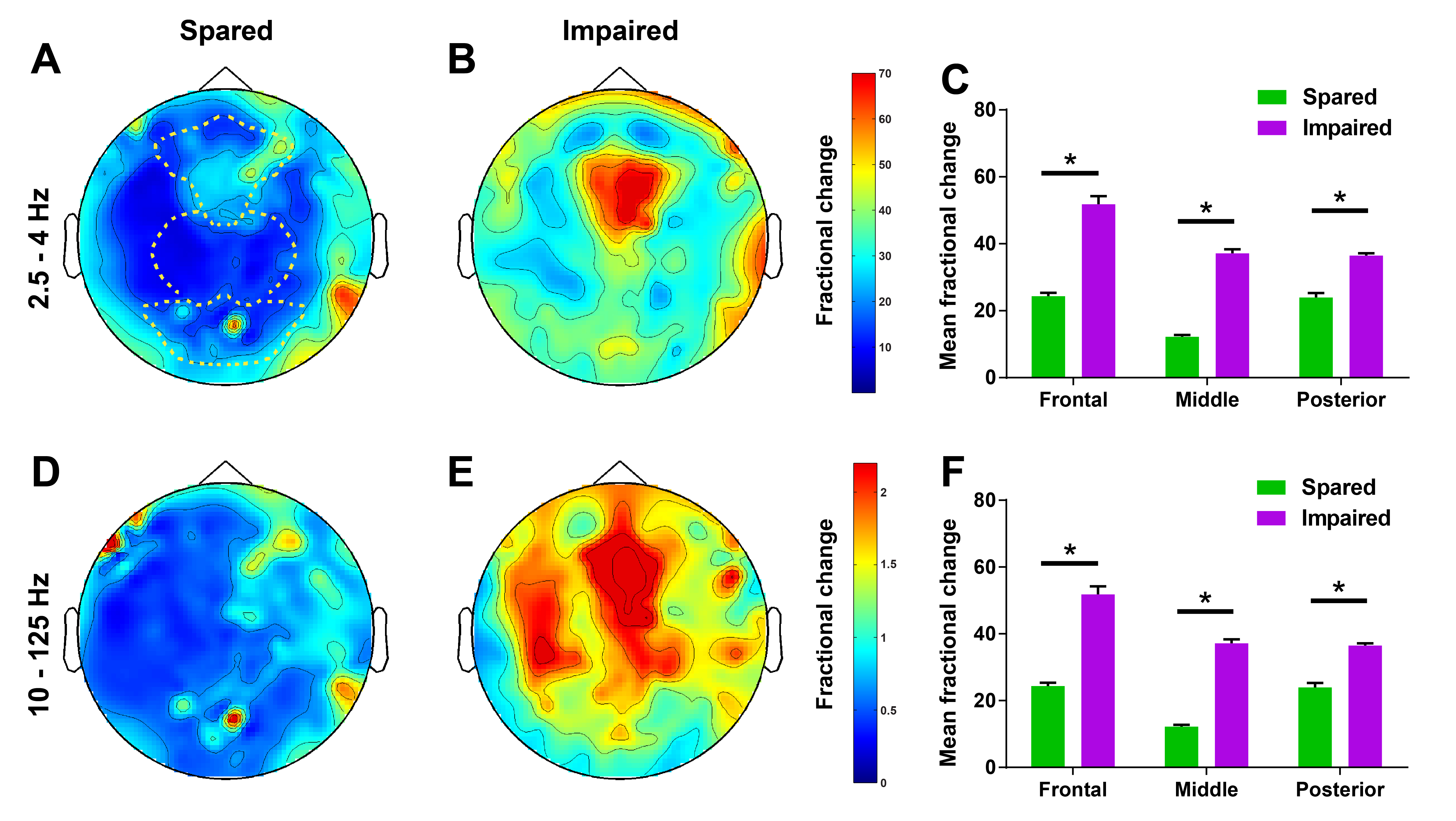
**

**Figure S17** Greater EEG amplitude in widespread brain regions during seizures with impaired performance on the RTT task. Confirmation of results from Figure 5 with single task subgroup analysis. **A-B.** Head maps of 256 channel high-density EEG power in the 2·5-4 Hz frequency range, representing the wave components of spike-wave discharges. Seizures with impaired task performance (B) demonstrate greater power in widespread regions compared to seizures with spared task performance (A). **C.** Greater 2·5–4 Hz power for impaired seizures was independent of electrode position (differences between means in fractional EEG power (95% CI): Frontal leads 27·5 (22·4 to 32·6); Middle leads 24·9 (22·5 to 27·4); Posterior leads 12·5 (9·6 to 15·4), p < 0·001 for all comparisons, paired t-test across electrodes in each region). **D-E.** Maps of EEG power in the 10-125 Hz frequency range (spike components of spike-wave discharges) for spared (D) and impaired (E) seizures. **F.** Greater 10–125 Hz EEG power for impaired seizures was independent of electrode position (differences between means in fractional EEG power (95% CI): Frontal leads 1·53 (1·39 to 1·66); Middle leads 1·23 (1·15 to 1·30); Posterior leads 0·56 (0·46 to 0·67), p < 0·001 for all comparisons, paired t-test across electrodes in each region). Color scale bars represent EEG power during seizures divided by baseline power prior to seizures (fractional power). Regions used for analysis in (C) and (F) Frontal, Middle, Posterior) are shown by dashed lines in (A). Error bars are SEM. n = 24 spared seizures in 5 patients, and 16 impaired seizures in 6 patients.

**Figure 18**

**
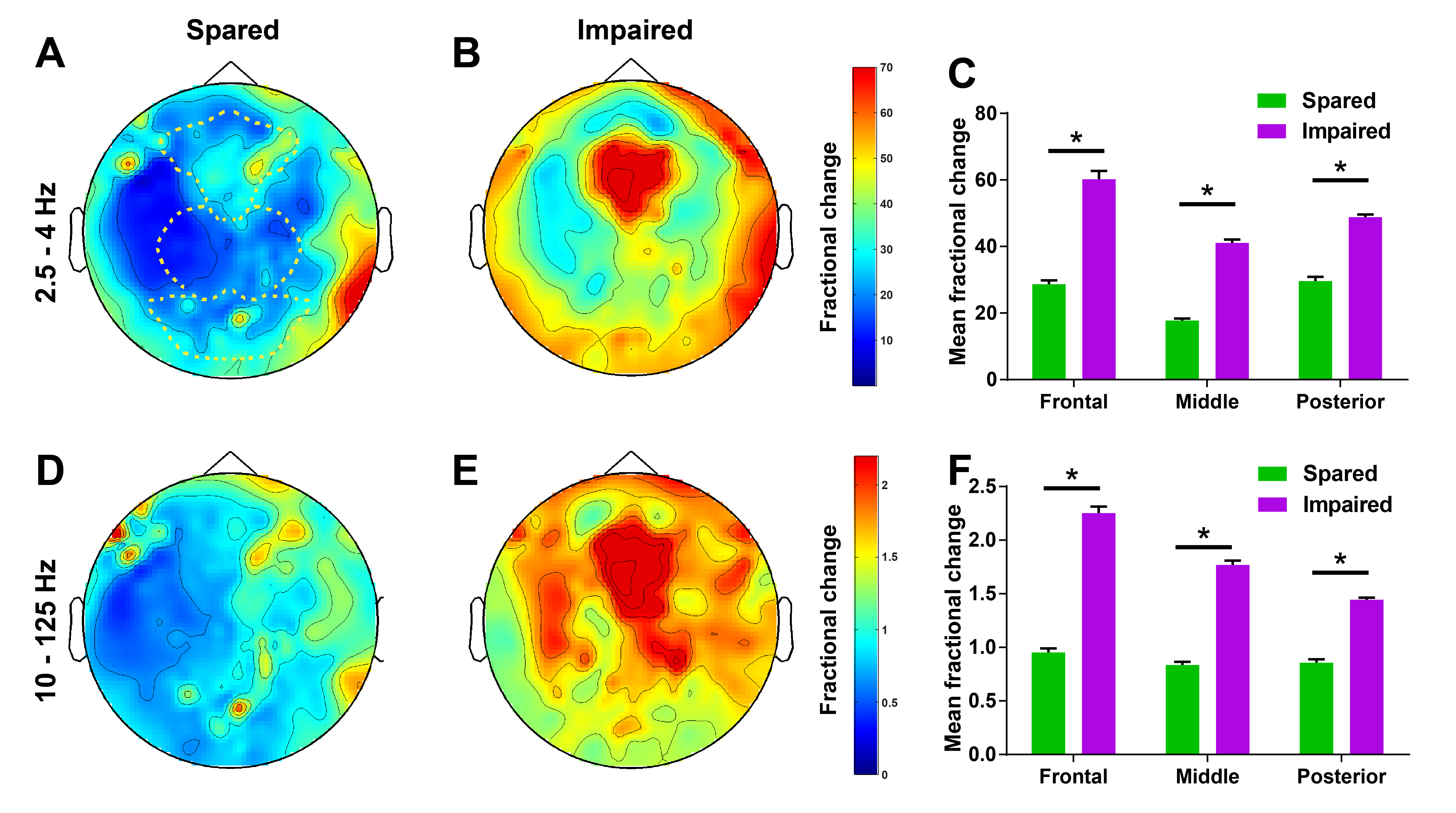
**

**Figure S18.** Confirmation of Figure 5 using broader definitions of behaviorally impaired and spared seizures. Comparison of EEG amplitudes for seizures with spared and impaired performance using all seizures captured with simultaneous behavior. For this analysis we defined ‘Impaired’ seizures as those with ≤50% correct response rates (instead of <25%), and ‘Spared’ seizures as those with >50% correct response rates to behavioral targets (instead of >75%). (A-B) Head maps of 256 channel high-density EEG power in the 2·5-4 Hz frequency range, representing the wave components of spike-wave discharges. Seizures with impaired task performance (B) demonstrate greater power in widespread regions compared to seizures with spared task performance (A). (C) This relationship was true independent of electrode position (differences between means in fractional EEG power (95% CI): Frontal leads 31·5 (26·2 to 36·7); Middle leads 23·4 (21·0 to 25·7); Posterior leads 19·2 (16·3 to 22·1), p < 0·001 for all comparisons, paired t-test across electrodes in each region). Similarly, (D-E) show maps of EEG power in the 10-125 Hz frequency range (spike components of spike-wave discharges) for spared (D) and impaired (E) seizures. (F) Greater EEG power for impaired seizures independent of electrode position (differences between means in fractional EEG power (95% CI): Frontal leads 1·30 (1·16 to 1·44); Middle leads 0·93 (0·84 to 1·03); Posterior leads 0·59 (0·52 to 0·66), p < 0·001 for all comparisons, paired t-test across electrodes in each region). Color scale bars are EEG power during seizures divided by baseline power prior to seizures (fractional power). Regions used for analysis in (C) and (F) (Frontal, Middle, Posterior) are shown by yellow dashed lines in (A). Error bars are SEM. n = 37 spared seizures in 7 patients, and 29 impaired seizures in 8 patients

**Figure 19**

**
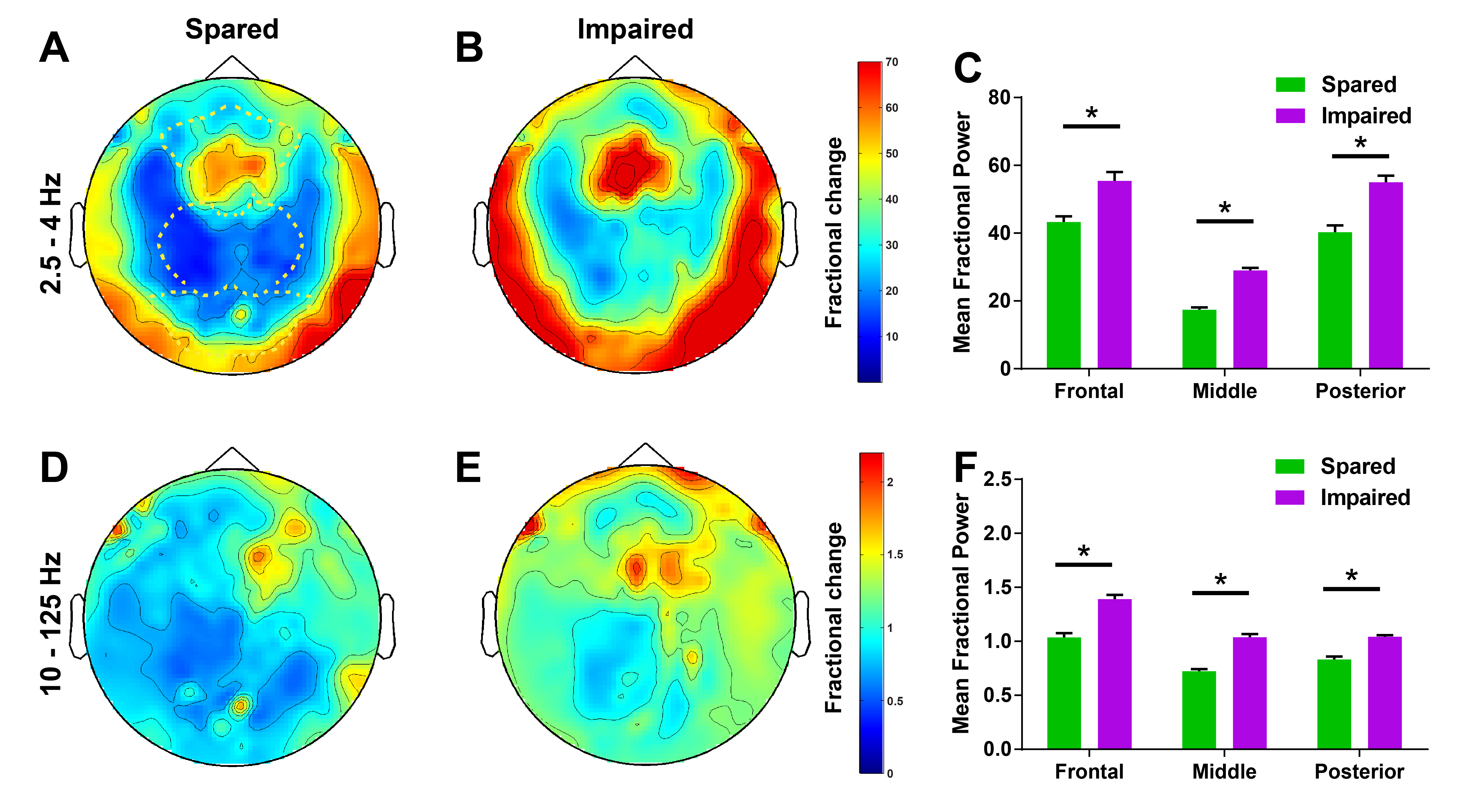
**

**Figure S19** Subgroup analysis of both impaired and spared seizures within the same patients. Greater EEG amplitude in widespread brain regions during seizures with impaired task performance in three patients who had seizures associated with both spared and impaired performance. (A-B) Head maps of 256 channel high-density EEG power in the 2·5-4 Hz frequency range, representing the wave components of spike-wave discharges. Seizures with impaired task performance (B) demonstrate greater power in widespread regions compared to seizures with spared task performance (A). (C) This relationship was true independent of electrode position (differences between means in fractional EEG power (95% CI): Frontal leads 12·1 (6·25 to 18·0); Middle leads 11·6 (9·62 to 13·5); Posterior leads 14·7 (9·23 to 20·2), p < 0·001 for all comparisons, paired t-test across electrodes in each region). Similarly, (D-E) show maps of EEG power in the 10-125 Hz frequency range (spike components of spike-wave discharges) for spared (D) and impaired (E) seizures. (F) Greater EEG power for impaired seizures independent of electrode position (differences between means in fractional EEG power (95% CI): Frontal leads 0·35 (0·24 to 0·46); Middle leads 0·31 (0·25 to 0·38); Posterior leads 0·21 (0·15 to 0·27), p < 0·001 for all comparisons, paired t-test across electrodes in each region). Color scale bars are EEG power during seizures divided by baseline power prior to seizures (fractional power). Regions used for analysis in (C) and (F) (Frontal, Middle, Posterior) are shown by yellow dashed lines in (A). Error bars are SEM. n = 28 spared and 11 impaired seizures in 3 patients.

| **Table S1. Demographics for patients with seizures** | | | | | | | | |
| --- | --- | --- | --- | --- | --- | --- | --- | --- |
| **Pt #** | **Sex** | **Age at scan (years)^1^** | **Age at onset**  **(years)** | **Treatment prior to study^2^** | **Number of spared^3^ seizures** | **Number of impaired^3^ seizures** | **Number of inter-mediate^3^ seizures** | **Number of other seizures^4^** |
| 1 | M | 12 | 12 | None^5^ | 1 | 0 | 3 | 3 |
| 2 | M | 11 | 7 | ESX | 2 | 0 | 3 | 2 |
| 3 | F | 12 | 7 | LTG | 19 | 44 | 64 | 232^9^ |
| 4 | F | 6 | 4 | None^6^ | 0 | 0 | 0 | 4 |
| 5 | M | 11 | 6 | LTG | 1 | 1 | 1 | 1 |
| 6 | F | 9 | 6 | LTG | 0 | 0 | 0 | 6 |
| 7 | M | 12 | 10 | LTG, VPA | 0 | 0 | 0 | 1 |
| 8 | M | 15 | 10 | LTG | 2 | 0 | 3 | 2 |
| 9 | F | 7 | 3 | LTG | 1 | 0 | 2 | 6 |
| 10 | F | 12 | 7 | LTG | 0 | 12 | 0 | 9 |
| 11 | M | 12 | 8 | VPA | 1 | 1 | 0 | 8 |
| 12 | M | 10 | 8 | LTG, FLB | 2 | 1 | 1 | 4 |
| 13 | F | 14 | 8 | ESX | 0 | 1 | 0 | 0 |
| 14 | M | 19 | 16 | None^7^ | 9 | 4 | 3 | 1 |
| 15 | F | 6 | 3 | ESX | 0 | 13 | 0 | 9 |
| 16 | F | 13 | 4 | ESX | 0 | 2 | 2 | 2 |
| 17 | F | 8 | 7 | VPA | 1 | 4 | 3 | 3 |
| 18 | F | 6 | 4 | None^6^ | 0 | 1 | 1 | 6 |
| 19 | M | 14 | 7 | LTG | 0 | 4 | 0 | 6 |
| 20 | F | 6 | 6 | None^7^ | 0 | 4 | 0 | 3 |
| 21 | M | 14 | 13 | None^8^ | 2 | 0 | 0 | 5 |
| 22 | F | 15 | 11 | None^6^ | 11 | 7 | 0 | 43 |
| 23 | F | 12 | 10 | FLB, LCS | 1 | 4 | 5 | 11 |
| 24 | F | 8 | 5 | ESX, VPA | 2 | 2 | 0 | 16 |
| 25 | F | 10 | 9 | None^7^ | 0 | 4 | 2 | 1 |
| 26 | M | 12 | 9 | VPA | 0 | 0 | 1 | 3 |
| 27 | M | 8 | 6 | ESX | 2 | 1 | 0 | 5 |
| 28 | F | 6 | 1 | ESX, LTG | 0 | 0 | 0 | 4 |
| 29 | F | 6 | 5 | LEV, ZNS | 3 | 4 | 2 | 7 |
| 30 | M | 7 | 4 | Modified Atkins diet | 14 | 11 | 6 | 12 |
| 31 | M | 7 | 6 | ESX, VPA | 0 | 2 | 2 | 0 |
| 32 | F | 8 | 5 | None^5^ | 0 | 1 | 0 | 13 |
| 33 | M | 10 | 10 | None^7^ | 0 | 5 | 2 | 9 |
| 34 | F | 12 | 11 | None^5^ | 4 | 1 | 1 | 37 |
| 35 | F | 10 | 2 | ESX | 3 | 3 | 4 | 9 |
| 36 | M | 6 | 6 | Ketogenic diet | 0 | 6 | 0 | 1 |
| 37 | F | 12 | 4 | Ketogenic diet | 53 | 17 | 11 | 84^9^ |
| 38 | F | 7 | 6 | ESX | 5 | 0 | 0 | 21 |
| 39 | F | 6 | 3 | LEV | 0 | 0 | 0 | 22 |
| **Total Seizures**  139 160 122 611  **Grand Total** 1032 | | | | | | | | |

**Table S1.** Demographics for patients with seizures during the study.

^1^Average age is shown for subjects with more than one visit. ^2^For subjects with more than one visit, only treatment at the first visit is shown. Medications were withheld for up to 48 hours prior to testing for all subjects. ^3^Spared seizures had correct responses to >75% of targets and impaired seizures had correct responses to <25% of targets on behavioral tasks. Intermediate seizures had correct response rates between 25% and 75%. ^4^Other seizures were obtained without behavioral data. These seizures were included in the analysis of generalizable fMRI network involvement during seizures (Figure 3). Reasons patients were off medications pre-study: ^5^Parental concerns for side effects; ^6^Parents did not observe seizures off medications; ^7^Newly diagnosed; ^8^Reason unknown. ^9^Patients 3 and 37 had more seizures compared to the others; however removing these two subjects from each of the analyses did not significantly change the results (data not shown). Abbreviations: ESX, ethosuximide; LTG, lamotrigine; VPA, valproic acid; FLB, felbamate; LCS, lacosamide; LEV, levetiracetam; ZNS, zonisamide.

**References**

1. ILAE. Proposal for revised classification of epilepsies and epileptic syndromes. Commission on Classification and Terminology of the International League Against Epilepsy. Epilepsia. 1989;30:389-99.

2. Killory BD, Bai X, Negishi M, Vega C, Spann MN, Vestal M, et al. Impaired attention and network connectivity in childhood absence epilepsy. Neuroimage. 2011;56(4):2209–17. [NIHMSID #289447].

3. Bai X, Guo J, Killory B, Vestal M, Berman R, Negishi M, et al. Resting Functional Connectivity between the Hemispheres in Childhood Absence Epilepsy. Neurology. 2011;76 (23):1960-7. [PMCID: 3109878].

4. Berman R, Negishi M, Vestal M, Spann M, Chung MH, Bai X, et al. Simultaneous EEG, fMRI, and behavior in typical childhood absence seizures. Epilepsia. 2010;51(10):2011-22.

5. Bai X, Vestal M, Berman R, Negishi M, Spann M, Vega C, et al. Dynamic time course of typical childhood absence seizures: EEG, behavior, and functional magnetic resonance imaging. J Neurosci. 2010;30(17):5884-93.

6. Berman R, Negishi M, Vestal M, Spann M, Chung M, Bai X, et al. Simultaneous EEG, fMRI, and behavioral testing in typical childhood absence seizures. Epilepsia. 2010;51(10):2011-22.

7. Mirsky AF, Van Buren JM. On the Nature of the "Absence" in Centrencephalic Epilepsy: A Study of some Behavioral, Electroencephalographic, and Autonomic Factors. Electroencephalogr Clin Neurophysiol. 1965;18:334-48.

8. Blumenfeld H. Consciousness and epilepsy: why are patients with absence seizures absent? Prog Brain Res. 2005;150:271-86. PubMed PMID: 16186030.

9. Carney PW, Masterton RA, Harvey AS, Scheffer IE, Berkovic SF, Jackson GD. The core network in absence epilepsy. Differences in cortical and thalamic BOLD response. Neurology. 2010;75(10):904-11.

10. Moeller F, Siebner HR, Wolff S, Muhle H, Boor R, Granert O, et al. Changes in activity of striato-thalamo-cortical network precede generalized spike wave discharges. Neuroimage. 2008;39(4):1839-49.

11. Negishi M, Abildgaard M, Laufer I, Nixon T, Constable RT. An EEG recording system with carbon wire electrodes for simultaneous EEG-fMRI recording. J Neurosci Methods. 2008;173(1):99-107.

12. Negishi M, Abildgaard M, Nixon T, Constable RT. Removal of time-varying gradient artifacts from EEG data acquired during continuous fMRI. Clinical Neurophysiology. 2004;115(9):2181-92.

13. Crowley M, Wu J, Molfese P, Mayes L. Social exclusion in middle childhood: Rejection events, slow-wave neural activity, and ostracism distress. Social Neuroscience. 2010;5(5):483-95.

14. Daly D, Pedley TA. Current Practice of Clinical Electroencephalography, 2nd Edition. New York: Raven Press. 1990.

15. Sadleir LG, Scheffer IE, Smith S, Carstensen B, Farrell K, Connolly MB. EEG features of absence seizures in idiopathic generalized epilepsy: impact of syndrome, age, and state. Epilepsia. 2009;50(6):1572-8.

16. Shimazono Y, Hirai T, Okuma T, Fukuda T, Yamamasu E. Disturbance of consciousness in petit mal epilepsy. Epilepsia. 1953;2:49-55.

17. Tuvo F. Contribution a l'etude des niveaux de conscience au cours des paroxysmes epileptiques infraclinique. Electroenceph and Clin Neurophysiol. 1958;10:715-8.

18. Browne TR, Penry JK, Porter RJ, Dreifuss FE. Responsiveness before, during and after spike-wave paroxysms. Neurology. 1974;24(7):659-65.

19. Krestel HE, Nirkko A, von Allmen A, Liechti C, Wettstein J, Mosbacher A, et al. Spike-triggered reaction-time EEG as a possible assessment tool for driving ability. Epilepsia. 2011 Oct;52(10):e126-9. PubMed PMID: 21883181. English.

20. Oppenheim AV, Schafer RW, Buck JR. Discrete-Time Signal Processing. Second ed. Upper Saddle River, NJ: Prentice-Hall, Inc.; 1999.

21. Huettel SA, Song AW, McCarthy G. Functional Magnetic Resonance Imaging. Third Edition ed. Sunderland, MA: Sinauer Associates, Inc.; 2014.

22. Power J, Barnes KA, Snyder AZ, Schlaggar BL, Petersen SE. Spurious but systematic correlations in functional connectivity MRI networks arise from subject motion. Neuroimage. 2012;59:2142-54.

23. Smyser C, Inder T, Shimony J, Hill J, Degnan A, Snyder A, et al. Longitudinal Analysis of Neural Network Development in Preterm Infants. Cereb Cortex. 2010;20:2852-62.

24. Handwerker D, Ollinger J, D'Esposito M. Variation of BOLD hemodynamic responses across subjects and brain regions and their effects on statistical analyses. Neuroimage. 2004;21:1639-51.

25. Gonzalez-Castillo J, Saad ZS, Handwerker DA, Inati SJ, Brenowitz N, Bandettini PA. Whole-brain, time-locked activation with simple tasks revealed using massive averaging and model-free analysis. Proc Natl Acad Sci U S A. 2012 Apr 3;109(14):5487-92. PubMed PMID: 22431587. English.

26. Rousseeuw P. Silhouettes: A graphical aid to the interpretation and validation of cluster analysis. J Comput Appl Math. 1987;20:53-65.

27. Salek-Haddadi A, Diehl B, Hamandi K, Merschhemke M, Liston A, Friston K, et al. Hemodynamic correlates of epileptiform discharges: An EEG-fMRI study of 63 patients with focal epilepsy. Brain Research. 2006;1088:148-66.

28. Kang JK, Benar C, Al-Asmi A, Khani YA, Pike GB, Dubeau F, et al. Using patient-specific hemodynamic response functions in combined EEG-fMRI studies in epilepsy. Neuroimage. 2003;20(2):1162-70.

29. Pugnaghi M, Carmichael DW, Vaudano AE, Chaudhary UJ, Benuzzi F, Di Bonaventura C, et al. Generalized spike and waves: effect of discharge duration on brain networks as revealed by BOLD fMRI. Brain Topogr. 2014 Jan;27(1):123-37. PubMed PMID: 23990340.

30. Friston KJ, Holmes AP, Worsley KJ. How many subjects constitute a study? Neuroimage. 1999;10(1):1-5.

31. Friston KJ, Holmes AP, Price CJ, Buchel C, Worsley KJ. Multisubject fMRI studies and conjunction analyses. Neuroimage. 1999 Oct;10(4):385-96. PubMed PMID: 10493897.

32. Monti MM. Statistical analysis of fMRI time-series: a critical review of the GLM approach. Front Hum Neurosci. 2011;5:28. PubMed PMID: 21442013. Pubmed Central PMCID: 3062970.

33. Worsley KJ, Marrett S, Neelin P, Vandal AC, Friston KJ, Evans AC. A unified statistical approach for determining significant signals in images of cerebral activation. Hum Brain Mapp. 1996;4(1):58-73. PubMed PMID: 20408186.

34. Brett M, Penny W, Kiebel S. Parameteric Procedures. In: Friston KJ, Kiebel S, Nichols TE, editors. Statistical Parametric Mapping: The Analysis of Functional Brain Images. London, UK: Elsevier Ltd.; 2007. p. 223-36.

35. Carney PW, Masterton RA, Flanagan D, Berkovic SF, Jackson GD. The frontal lobe in absence epilepsy: EEG-fMRI findings. Neurology. 2012 Apr 10;78(15):1157-65. PubMed PMID: 22459682. English.
